# Highly conserved extended haplotypes of the major histocompatibility complex and their relationship to multiple sclerosis susceptibility

**DOI:** 10.1371/journal.pone.0190043

**Published:** 2018-02-13

**Authors:** Douglas S. Goodin, Pouya Khankhanian, Pierre-Antoine Gourraud, Nicolas Vince

**Affiliations:** 1 Department of Neurology, University of California, San Francisco, CA, United States of America; 2 Center for Neuro-engineering and Therapeutics, University of Pennsylvania, Philadelphia, PA, United States of America; 3 Centre de Recherche en Transplantation et Immunologie UMR 1064, INSERM, Université de Nantes, Nantes, France; 4 Institut de Transplantation Urologie Néphrologie (ITUN), CHU Nantes, Nantes, France; University of Alabama at Birmingham, UNITED STATES

## Abstract

**Objective:**

To determine the relationship between highly-conserved extended-haplotypes (CEHs) in the major histocompatibility complex (MHC) and MS-susceptibility.

**Background:**

Among the ~200 MS-susceptibility regions, which are known from genome-wide analyses of single nucleotide polymorphisms (SNPs), the MHC accounts for roughly a third of the currently explained variance and the strongest MS-associations are for certain Class II alleles (e.g., HLA-DRB1*15:01; HLA-DRB1*03:*01;* and HLA-DRB1*13:*03*), which frequently reside on CEHs within the MHC.

**Design/Methods:**

Autosomal SNPs (441,547) from 11,376 MS cases and 18,872 controls in the WTCCC dataset were phased. The most significant MS associated SNP haplotype was composed of 11 SNPs in the MHC Class II region surrounding the *HLA-DRB1 gene*. We also phased alleles at the *HLA-A*, *HLA-C*, *HLA-B*, *HLA-DRB1*, and *HLA-DQB1* loci. This data was used to probe the relationship between CEHs and MS susceptibility.

**Results:**

We phased a total of 59,884 extended haplotypes (*HLA-A*, *HLA-C*, *HLA-B*, *HLA-DRB1*, *HLA-DQB1* and SNP haplotypes) from 29,942 individuals. Of these, 10,078 unique extended haplotypes were identified. The 10 most common CEHs accounted for 22% (13,302) of the total. By contrast, the 8,446 least common extended haplotypes also accounted for approximately 20% (12,298) of the total. This extreme frequency-disparity among extended haplotypes necessarily complicates interpretation of reported disease-associations with specific HLA alleles. In particular, the HLA motif HLA-DRB1*15:01~HLA-DQB1*06:*02* is strongly associated with MS risk. Nevertheless, although this motif is almost always found on the *a1* SNP haplotype, it can rarely be found on others (e.g., *a27* and *a36*), and, in these cases, it seems to have no apparent disease-association (OR = 0.7; CI = 0.3–1.3 and OR = 0.7; CI = 0.2–2.2, respectively). Furthermore, single copy carriers of the *a1* SNP-haplotype without this HLA motif still have an increased disease risk (OR = 2.2; CI = 1.2–3.8). In addition, even among the set of CEHs, which carry the Class II motif of HLA-DRB1*15:01~HLA-DQB1*06:*02~a1*, different CEHs have differing strengths in their MS-associations.

**Conclusions:**

The MHC in diverse human populations consists, primarily, of a very small collection of very highly-selected CEHs. Our findings suggest that the MS-association with the HLA-DRB1*15:01~HLA-DQB1*06:*02* haplotype may be due primarily to the combined attributes of the CEHs on which this particular HLA-motif often resides.

## Introduction

The basis of genetic susceptibility to multiple sclerosis (MS) is complex [1–3]. Thus, currently, there are over 200 MS associated common risk variants in different genomic regions that have been identified by genome wide association screens (GWAS) comparing MS patients to controls [4–12]. These GWAS studies typically evaluate the disease associations for ~500,000 single nucleotide polymorphisms (SNPs) scattered throughout the genome [4–12]. Despite the large number of genetic associations defined by these increasingly available GWAS studies, several alleles of the human leukocyte antigens (HLA), located in the major histocompatibility complex (MHC) on the short arm of chromosome 6 (6p21.3), were identified more than four decades ago. The most prominent of these HLA associations (by far) is with the HLA-DRB1*15:*01* allele, which typically has an odds ratio (OR) of more than three for heterozygotes and more than six for homozygotes [9, 13–20]. Also, other alleles at the *DRB1* locus (e.g., HLA-DRB1*03:*01* and HLA-DRB1*13:*03*) are known to be associated with an increased risk of getting MS [1, 11, 21]. However, even with the large number of defined genetic associations with MS, most of the genetic risk in MS remains unexplained. In addition, as shown in *Figure A in S3 File,* the large majority of the population does not even belong to the subgroup of individuals who are “genetically susceptible” to getting MS [3]. Observations such as these have created a so-called “heritability gap”. Such a gap is a common finding in many complex genetic disorders [1, 2] and is likely due (at least in part) to the phenomenon of “synthetic association” [22], in which a reported association is simply tagging a genomic region rather than identifying a causal variant. Indeed, both single SNPs and single alleles can be associated with several haplotypes sometimes spread over a considerable genetic distance [23–34]. For example, despite the apparently well-established association of MS susceptibility with the HLA-DRB1*15:*01* allele, this association might be due to a synthetic association [18, 19]. Moreover, as demonstrated in *Figure A in S3 File*, even for the HLA-DRB1*15:*01* allele, the large majority of its carriers do not even belong to the subset of individuals who are “genetically susceptible” to getting MS [3].

Some of the haplotypes in the MHC region are highly conserved extended haplotypes (CEHs), which span more than 2.7 megabases (mb) [23–28, 30, 32–36]. These CEHs exist even though the MHC region encompasses several recombination hotspots and the region as a whole has an average recombination rate of ~0.4 centimorgans (cM) per mb [27, 34, 37, 38]. Proposed mechanisms to account for this kind of extended linkage are: “frozen blocks” of DNA, preservation of ancestral lineages, haplotype-specific suppression of recombination / mutation in parts of the MHC region, or some form of balancing evolution, in which heterozygosity is favored [24, 39–43]. Several of these CEHs include HLA-DRB1*15:*01*, HLA-DRB1*03:*01*, HLA-DRB1*13:*03*, or other alleles. For example, the haplotypes:

HLA‑A*0101∼HLA‑C*07:01∼HLA‑B*08:01∼HLA‑DRB1*03:01∼HLA‑DQB1*02:01

HLA‑A*03:01∼HLA‑C*07:02∼HLA‑B*07:02∼HLA‑DRB1*15:01∼HLA∼DQB1*06:02,

and:

HLA‑A*25:01∼HLA‑C*12:03∼HLA‑B*18:01∼HLA‑DRB1*15:01∼HLA‑DQB1*06:02

have been consistently observed in Caucasian populations [23–28, 32, 35, 38]. Necessarily, the existence of such CEHs in the MHC region complicates the interpretation of the disease association with any specific HLA allele. We recently explored a method for reducing the size of the heritability gap by analyzing SNP haplotypes (rather than single SNPs) throughout the genome [32]. In addition to improving significantly the explained genetic risk, this method also provides an opportunity to explore in greater depth the genetic associations of the MHC reported previously.

**Fig 1:**
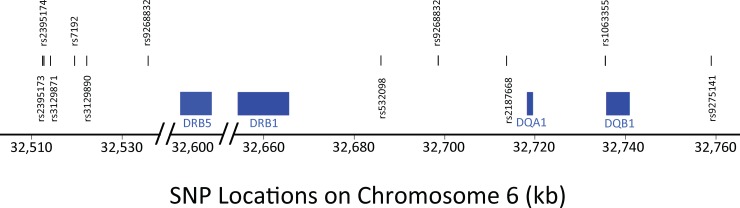
Location of the 11 SNPs in the haplotype surrounding the Class II DRB1 gene on chromosome 6 (6p21.3), which had the greatest disease association of any SNP haplotype in the region (see text). The blue rectangles span the regions from the start to the stop points of the Class II genes: *HLA*-*DRB5*, *HLA*-*DRB1*, *HLA*-*DQA1*, and *HLA*-*DQB1*. The centromere of Chromosome 6 lies to the right of this portion of 6p21.3.

**Fig 2:**
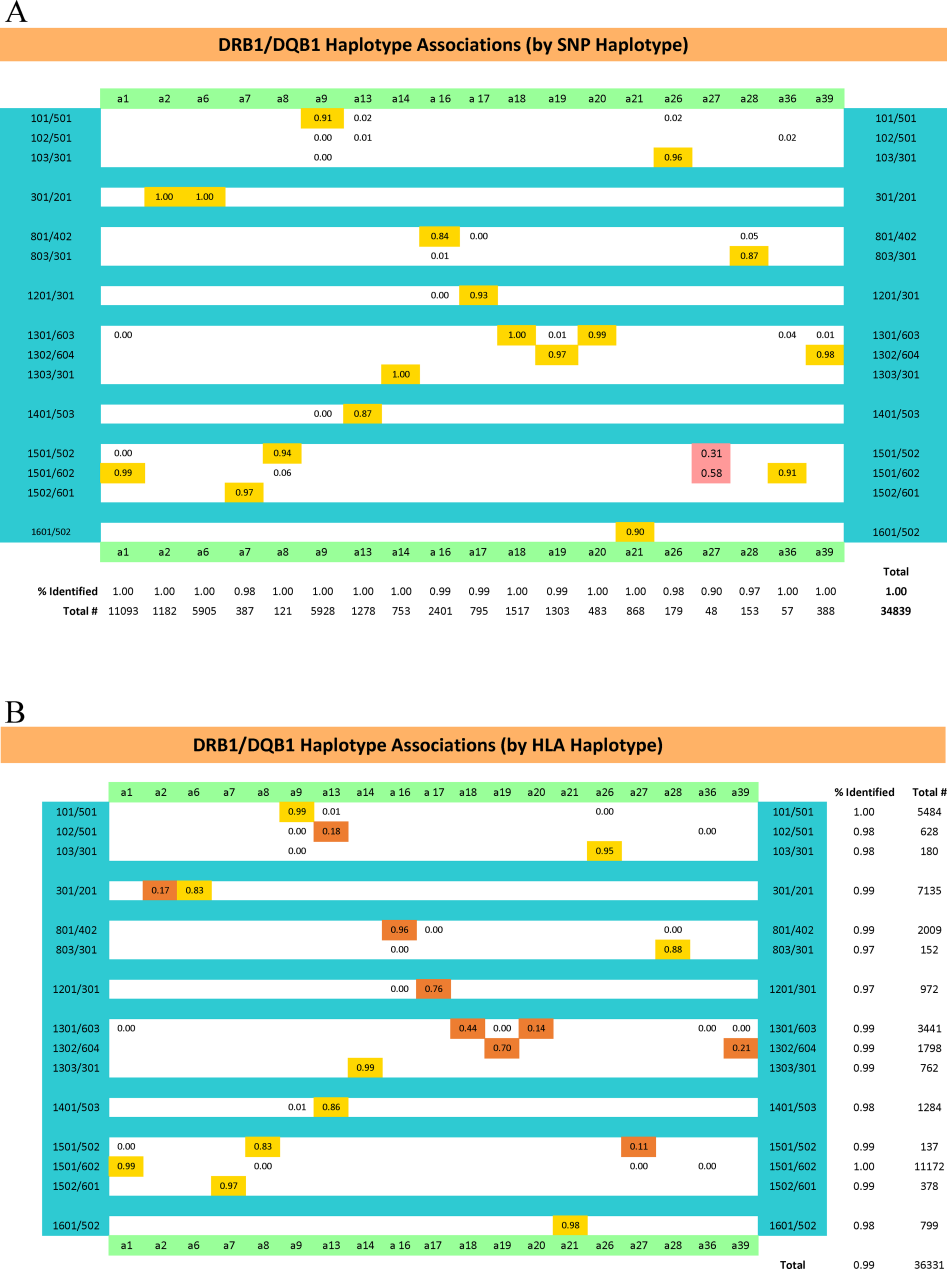
The HLA haplotype/SNP haplotype associations–both by SNP haplotype (A) and also by HLA haplotype (B)–for selected SNP haplotypes (some of which are presented in Table 1). Other haplotypes not presented also had very specific haplotype associations [32].

For example, using the Wellcome Trust Case Control Consortium dataset (WTCCC), we found an 11-SNP haplotype in the MHC region, which had the greatest MS disease association of any, and which we labeled the *a1* SNP haplotype (OR [single copy] ≈ 3; p<10^−300^) [29, 30]. This SNP haplotype represents a specific string of 11 SNPs spanning a total of 246.3 kilobases (kb) surrounding the *HLA-DRB1* gene (Fig 1) and includes the SNPs (*rs2395173*, *rs2395174*, *rs3129871*, *rs7192*, *rs3129890*, *rs9268832*, *rs532098*, *rs17533090*, *rs2187668*, *rs1063355*, *and rs9275141*). These 11 SNPs define 174 haplotypes in this region (e.g., Table 1), with each SNP haplotype having its own Class II HLA haplotype specificity (e.g., Table 1; Fig 2). As with other previously reported SNP “hits” in this genomic region [9, 13–17], the *a1* SNP haplotype is tightly coupled to the MHC Class II haplotype of HLA-DRB1*15:01~HLA-DQB1*06:*02*. In the present paper, we have analyzed the haplotype structure of the MHC (including both HLA alleles and SNP haplotypes) to better understand the specific genetic relationship of this genomic region to MS.

**Table 1:** Selected SNP haplotypes in the Class II region of chromosome 6^†^.

	SNP	HLA		
Name	Haplotype	Association	WTCCC	EPIC
*a1*	10110100010	*HLA*-DRB1*15:*01~* *HLA*-DQB1*06:*02*	0.12	0.11
*a2*	00000000100	*HLA*-DRB1*03:*01~* *HLA*-DQB1*02:*01*	0.02	0.02
*a3*	00000010001	*multiple haplotypes*^*††*^	0.19	0.21
*a4*	00000000001	*HLA*-DRB1*11:*01~* *HLA*-DQB1*03:*01*	0.11	0.13
*a5*	10100010001	*HLA*-DRB1*07:*01~* *HLA*-DQB1*02:*02*	0.09	0.08
*a6*	01011100100	*HLA*-DRB1*03:*01~* *HLA*-DQB1*02:*01*	0.10	0.09
*a8*	10110100011	*HLA*-DRB1*15:*01~* *HLA*-DQB1*05:*02*	0.00	0.00
*a9*	01000001010	*HLA*-DRB1*01:*01~* *HLA*-DQB1*05:*01*	0.11	0.11
*a11*	00000010010	*HLA*-DRB1*13:*01~* *HLA*-DQB1*06:*03*	0.02	0.03
*a14*	10111111001	*HLA*-DRB1*13:*03~* *HLA*-DQB1*03:*01*	0.01	0.01
*a27*	10100100011	*two haplotypes*^*§*^	0.00	0.00
*a34*	10111100010	*HLA*-DRB1*15:*01~* *HLA*-DQB1*06:*02*^*§§*^	0.00	0.00
*a36*	10100100010	*HLA*-DRB1*15:*01~* *HLA*-DQB1*06:*02*^*§§*^	0.00	0.00
*a43*	00000100010	*HLA*-DRB1*15:*01~* *HLA*-DQB1*06:*02*	0.00	0.00

† The "Name" is arbitrary and indicates the order of haplotype identification in the EPIC dataset [29, 30]. The SNP haplotype represents the haplotypes identified using the set of 11 SNPs shown in Fig 1 and provided in text. The number “0” indicates the presence of the major allele and the number “1” indicates the presence of the minor allele (in the control population) at the particular SNP location. Only 14 selected SNP-haplotypes (of the 174 present in the WTCCC) are listed. Haplotype frequencies found in two independent datasets (EPIC and WTCCC) are shown [29, 30]. Frequencies are provided to 2 significant digits after the decimal. Those listed as (0.00) were less than 0.005. Each of the 174 haplotypes had very specific associations with specific Class II haplotypes. For example, each of the associations (shown in the Table) of specific SNP-haplotypes with specific HLA haplotypes were highly significant. Almost all had of p-value (by Chi square analysis) of (p<10^−300^). The only two exceptions to this were for *HLA*-DRB1*07:*01~HLA*-DQB1*02:*02~a3* (p<10^−151^) and for *HLA*-DRB1*15:*01~HLA*-DQB1*06:*02~a34* (p<10^−290^). Moreover, both the EPIC and the WTCCC datasets had the same Class II HLA associations with the different SNP-haplotypes.

†† In both EPIC and the WTCCC, *a3* was equally associated with four HLA haplotypes: *HLA*-DRB1*04:*01~HLA*-DQB1*03:*01*, *HLA*-DRB1*04:*01~HLA*-DQB1*03:*02*, *HLA*-DRB1*04:*04~HLA*-DQB1*03:*02*, and *HLA*-DRB1*07:*01~HLA*-DQB1*02:*02*.

§ In both EPIC and WTCCC, *a27* is associated with two haplotypes: *HLA*-DRB1*15:*01~HLA*-DQB1*06:*02*, and *HLA*-DRB1*15:*01~HLA*-DQB1*05:*02*,. In WTCCC, 58% (28/48) were *HLA*-DRB1*15:*01~HLA*-DQB1*06:*02*, whereas, in EPIC, none of the five *a27* SNP haplotypes were associated with this particular HLA haplotype.

§§ The single example of the *a34* SNP haplotype in EPIC was associated with the *HLA*-DRB1*15:*01~HLA*-DQB1*06:*02* HLA haplotype. No examples of the *a36* SNP haplotype were present in EPIC who also had HLA information.

## Results

### Highly conserved haplotypes of the MHC

**Fig 3:**
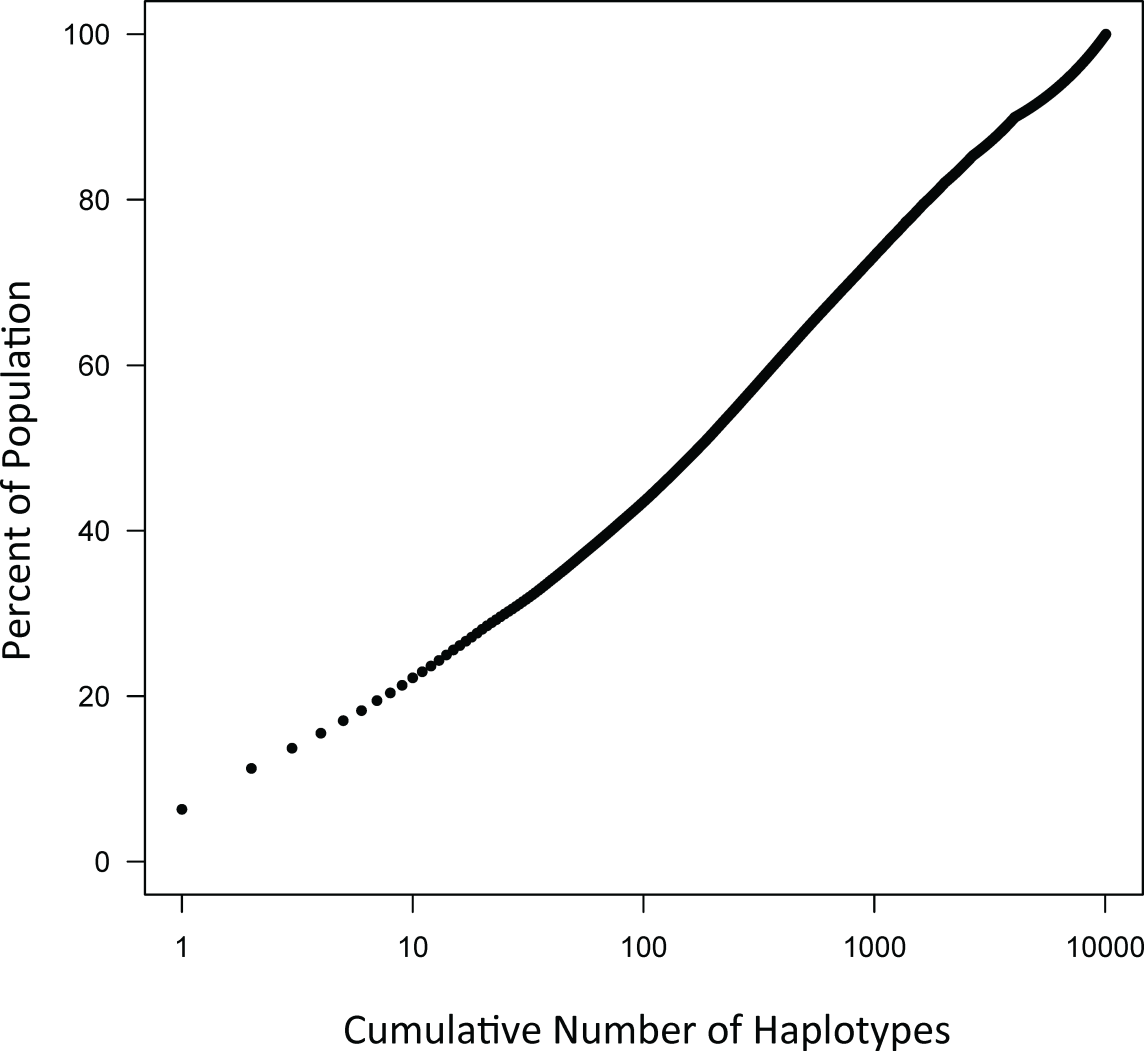
The WTCCC dataset consists of 59,884 haplotypes, of which 10,078 represent different (unique) combinations of the 5 HLA alleles and the SNP haplotypes (see text). For the purpose of this graph, these unique haplotypes (CEHs) have been sorted according to their descending frequency of occurrence in the WTCCC dataset. The cumulative number of unique haplotypes (beginning with the highest frequency haplotype) has been plotted against the percentage of total number of haplotypes in the population. As can be appreciated from the graph, the large majority (~80%) of the different CEHs have only a very low frequency, whereas 80% of the haplotypes in the population are accounted for by only small number of very common CEHs (i.e., ~10 haplotypes).

**Fig 4:**
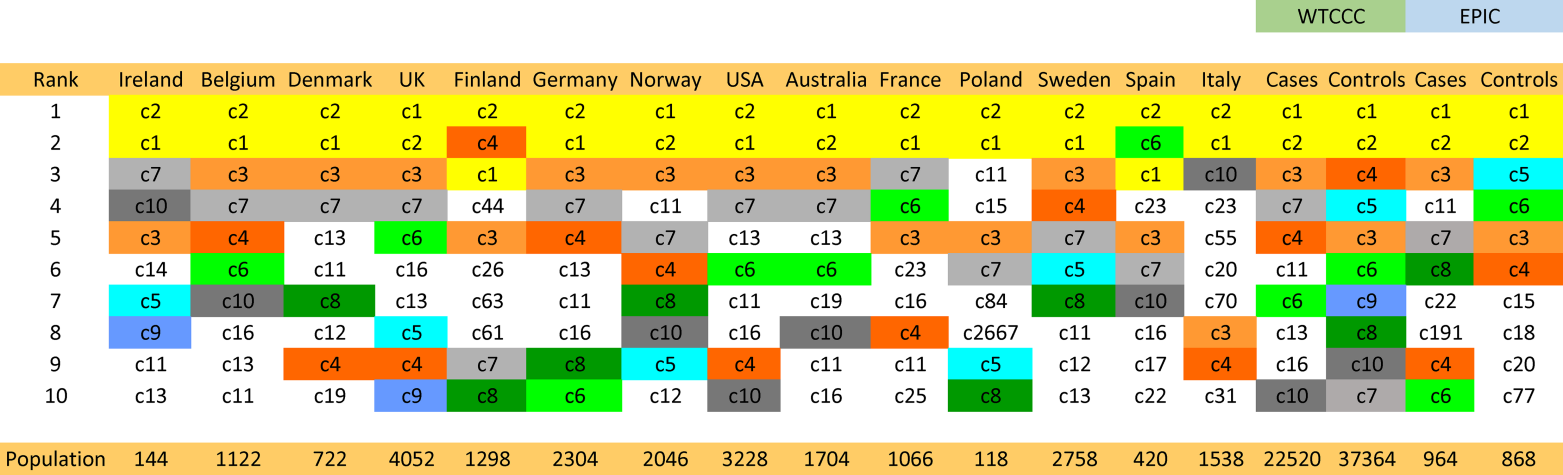
Rank order for the 10 most common extended haplotypes for the entire WTCCC dataset (labeled: *c1* to *c10078*; in descending order of frequency). The rank order of the haplotypes for each participating region are shown separately (see *S1 Table* for definitions of those haplotypes, which have been colored in the figure based on the overall 10 most common haplotypes in the WTCCC). Regions are ordered (from left to right) based on the descending frequency of the *c2* haplotype. Only cases are available for all regions. Nevertheless, both the complete WTCCC (Case and Control) and the EPIC (Case and Control) populations are also included for comparison.

Some of the CEHs in the MHC region, which are highly conserved, involve both Class I and Class II loci [24–38]. The different combinations of alleles at three Class I loci (*HLA*-*A*, *HLA*-*B*, and *HLA*-*C*) and two Class II loci (*HLA*-*DRB1* and *HLA*-*DQB1*) together with a specific 11-SNP haplotype represent more than 4 billion possible unique haplotypes spanning a genomic distance of 2.7 mb. Despite this huge number of possibilities, the frequency distribution for these extended haplotypes in the WTCCC is definitely non-Gaussian, with many very rare haplotypes together with a small number of very common haplotypes (e.g., Fig 3; *Figure B in S3 File*; *S1 Table; S2 Table*). Thus, there were just 10,078 unique haplotypes represented within the 29,942 individuals of the WTCCC accounting for 59,884 total observed haplotypes. Of these, 13,302 (22%) were accounted for by the most common 10 CEHs, 30% by the most common 25 CEHs, 48% by the 146 CEHs with 50 or more representations in the WTCCC, and 71% by the most common 810 CEHs (*S1 Table*). On the other end, 6,016 (60%) of the unique extended haplotypes were observed only once in the WTCCC dataset. An additional 1,397 (14%) had only 2 representations so that 7,413 (74%) of the unique haplotypes had two or fewer representations. However, these 74% of the unique haplotypes accounted for only 8,810 (15%) of the total number of observed haplotypes in the WTCCC dataset. Consequently, there exists a small set of very common CEHs, which have been strongly selected (see *S2 File)*, and which, nonetheless, have notably different compositions in different populations, even among relatively nearby geographic regions (Fig 4; *S1 and S2 Tables*). Moreover, there also appears to be a substantial amount of mixing between specific Class I and Class II motifs (see *S1 File*).

In addition, the prevalence of individuals in the WTCCC who were homozygous for these CEHs was increased relative to expectations (expected = 269; observed = 383; z = 6.97; p<10^−11^). Such an increase was found for both the cases (expected = 152; observed = 208; z = 4.59; p<10^−5^) and the controls (expected = 138; observed = 175; z = 3.13; p = 0.0018).

### Haplotype associations with MS susceptibility

The fact that much (possibly most) of the MHC is composed of a small group of CEHs necessarily complicates the interpretation of any disease associations previously reported for specific alleles such as *HLA*-DRB1*15:*01*, *HLA*-DRB1*03:*01*, and *HLA*-DRB1*13:*03* [1, 9, 13–17, 19, 21, 29, 30]. For example, it is unclear to what extent the effect of *HLA*-DRB1*15:*01* on disease susceptibility can be separated from an effect of the full CEHs (comprising both the 5 HLA alleles and the SNP haplotypes) on which this allele resides. To investigate this, we undertook two alternative approaches. First, we examined the disease association of different CEHs, which contained *HLA*-DRB1*15:*01~HLA*-DQB1*06:*02~a1*, *HLA*-DRB1*03:*01~HLA*-DQB1*02:*01~a2*, *HLA*-DRB1*03:*01~HLA*-DQB1*02:*01~a6*, or *HLA*-DRB1*13:*03~HLA*-DQB1*03:*01~a14*. Second, we examined the disease associations for haplotypes that either contained these same Class II HLA motifs but a different SNP haplotype motif or contained the same SNP haplotype motif but a different Class II HLA motif.

#### HLA-DRB1*15:01~HLA-DQB1*06:02

The *HLA*-DRB1*15:*01~HLA*-DQB1*06:*02* haplotype is very closely associated with the (*a1*) SNP haplotype; 99% of all (*a1*)-carriers also carry *HLA*-DRB1*1501~HLA-DQB1*0602 and the reciprocal statement is true as well (Fig 2). The disease associations of all CEHs containing *HLA*-DRB1*15:*01~HLA*-DQB1*06:*02~a1* with 50 or more representations in the WTCCC dataset are shown in Table 2. Each of these extended haplotypes is significantly associated with an increased disease risk (Table 2). However, for several of them, the magnitude of the association with disease risk varied significantly (*Figure C in S3 File*). Indeed, for example, the disease-association for haplotype (*c2*) was significantly greater that for than both the (*c3*) and the (*c11*)) haplotypes (*Figure C in S3 File*). By contrast, the haplotype (*c3*) had a significantly smaller disease-association than that of several other haplotypes (*Figure C in S3 File*). Especially notable, however, was haplotype (*c282*), consisting of *HLA*-A*03:*01~HLA*-C*15:*02~HLA*-B*51:*01~HLA*-DRB1*15:*01~HLA*-DQB1*06:*02~a1*, which had an extremely strong disease association (OR = 20.3; CI = 6.1− 67.3; p<10^−11^), and which differed significantly from every other haplotypes with the exception of the (*c173*) haplotype (*Figure C in S3 File*). However, regardless of the fact that the magnitude of disease association depends upon the particular CEH, on which the *HLA*-DRB1*15:*01~HLA*-DQB1*06:*02~a1* motif resides, some disease risk seems to be attributable to the *HLA*-DRB1*15:*01~HLA*-DQB1*06:*02~a1* haplotype by itself because the disease risk is still significantly increased for those individuals who both carry this complete Class II motif and, yet, whose full CEH has only a single representation in the WTCCC (OR = 3.0; CI = 2.7−3.4; p<10^−10^).

**Table 2:** Common *a1*-containing extended haplotypes in the WTCCC^††^.

	HLA Haplotype			
Name^†^	*A~C~B~DRB1~DQB1~SNP*	Frequency	OR*	p-value**
*c2*^*§*^	*03*:*01~07*:*02~07*:*02~15*:*01~06*:*02~a1*	2961	3.2 (3.0–3.5)	< E-168
*c3*^*§*^	*02*:*01~07*:*02~07*:*02~15*:*01~06*:*02~a1*	1465	2.2 (2.0–2.5)	< E-38
*c6*	*24*:*02~07*:*02~07*:*02~15*:*01~06*:*02~a1*	728	2.8 (2.4–3.3)	< E-36
*c11*	*25*:*01~12*:*03~18*:*01~15*:*01~06*:*02~a1*	440	3.9 (3.1–4.8)	< E-39
*c13*	*01*:*01~07*:*02~07*:*02~15*:*01~06*:*02~a1*	405	3.4 (2.7–4.2)	< E-29
*c16*	*01*:*01~07*:*01~08*:*01~15*:*01~06*:*02~a1*	320	3.7 (2.9–4.8)	< E-27
*c19*	*02*:*01~05*:*01~44*:*02~15*:*01~06*:*02~a1*	289	2.1 (1.6–2.7)	< E-7
*c22*	*11*:*01~07*:*02~07*:*02~15*:*01~06*:*02~a1*	229	2.5 (1.9–3.4)	< E-9
*c28*	*01*:*01~06*:*02~37*:*01~15*:*01~06*:*02~a1*	178	4.5 (3.2–6.3)	< E-20
*c44*	*31*:*01~07*:*01~18*:*01~15*:*01~06*:*02~a1*	135	2.9 (2.0–4.2)	< E-9
*c50*	*02*:*01~03*:*04~40*:*01~15*:*01~06*:*02~a1*	124	3.1 (2.0–4.7)	< E-7
*c58*	*02*:*01~03*:*03~15*:*01~15*:*01~06*:*02~a1*	105	3.2 (2.1–5.0)	< E-7
*c78*	*29*:*02~16*:*01~44*:*03~15*:*01~06*:*02~a1*	84	3.7 (2.2–6.1)	< E-7
*c87*	*31*:*01~07*:*02~07*:*02~15*:*01~06*:*02~a1*	73	3.4 (2.0–5.6)	< E-6
*c91*	*26*:*01~07*:*02~07*:*02~15*:*01~06*:*02~a1*	71	2.6 (1.6–4.3)	< E-3
*c108*	*32*:*01~07*:*02~07*:*02~15*:*01~06*:*02~a1*	64	3.1 (1.8–5.4)	< E-4
*c116*	*31*:*01~15*:*02~51*:*01~15*:*01~06*:*02~a1*	60	4.3 (2.4–7.9)	< E-6
*c120*	*03*:*01~04*:*01~35*:*01~15*:*01~06*:*02~a1*	58	4.5 (2.5–8.1)	< E-7
*c125*	*11*:*01~03*:*03~55*:*01~15*:*01~06*:*02~a1*	57	1.9 (1.1–3.3)	< 0.05
*c128*	*68*:*01~07*:*04~44*:*02~15*:*01~06*:*02~a1*	55	2.9 (1.6–5.1)	< E-3
*c132*	*01*:*01~06*:*02~57*:*01~15*:*01~06*:*02~a1*	54	1.8 (1.0–3.3)	< 0.05
*c139*^*§§*^	*02*:*01~03*:*04~15*:*01~15*:*01~06*:*02~a1*	52	3.2 (1.6–6.3)	< E-3
*c140*	*11*:*01~15*:*02~51*:*01~15*:*01~06*:*02~a1*	52	3.3 (1.7–6.4)	< E-3
*c143*	*68*:*01~07*:*02~07*:*02~15*:*01~06*:*02~a1*	51	3.0 (1.6–5.6)	< E-3
*c173*	*23*:*01~07*:*01~49*:*01~15*:*01~06*:*02~a1*	43	5.5 (2.8–10.9)	< E-7
*c282*	*03*:*01~15*:*02~51*:*01~15*:*01~06*:*02~a1*	29	20.3 (6.1–67.3)	< E-11

†† *a1* containing haplotypes with ≥ 50 representations in the WTCCC. Two additional haplotypes with fewer representations are also shown.

† Arbitrary name for haplotype (sorted in descending order of frequency) for the entire WTCCC population.

* Odds ratio (OR) of disease for individuals having 1 copy of the listed haplotype compared to having no other copies of the *HLA*-DRB1*15:*01~HLA*-DQB1*06:*02~a1* Class II haplotype (95% CI range in parenthesis). A Bonferroni correction for the number of haplotypes with 50 or more representations (146) would require a significance level of p<3*E-4.

** Significance of the association between having 1 copy of the specific allele and the disease (MS) compared to having no copies. The p-values are expressed in scientific notation as powers of 10 (E). All observations with (*p<0*.*001*) still demonstrated a statistically significant effect even after adjustment for population stratification, geographic stratification, and gender. Moreover, including each of these haplotypes in the same regression equation demonstrated that each of the listed CEHs was independently associated with having MS.

§ These two haplotypes also differed (non-significantly) in their disease-association for having two copies of each allele compared to having no copies of the *HLA*-DRB1*15:*01~HLA*-DQB1*06:*02~a1* Class II haplotype. Thus, these ORs are

For *c2*: OR [two copies] = 5.8 (3.4–9.9)

And, for *c3*: OR [two copies] = 2.7 (1.3–5.5)

§§ The Class I and Class II portions of each listed haplotype were significantly associated with each other beyond the Bonferroni-adjusted level of significance. The only exception to this rule was for the haplotype *c139*. In this case, the association had a p-value of: p = 4.42*E−8

**Fig 5:**
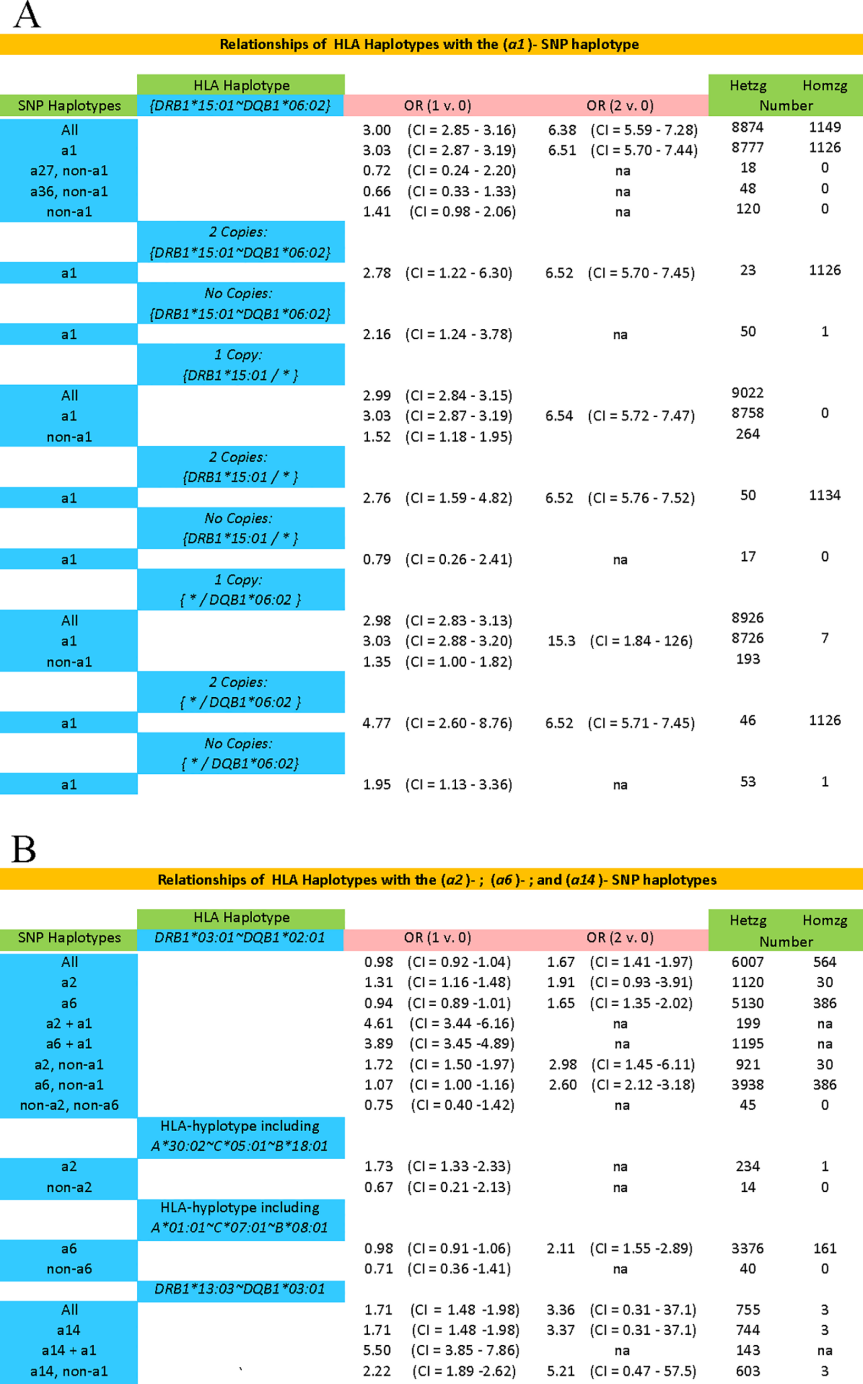
Disease-associations for the different SNP-haplotype combinations with the Class II HLA haplotypes of: (A) DRB1*1501~DQB1*0602 and: (B) DRB1*03:01~DQB1*02:01 & DRB1*13:03~DQB1*03:*01*. The odds ratios (OR) are given comparing cases to controls with regard to carrying either one or two copies of the risk-haplotype as opposed to carrying zero copies. In these circumstances, the disease association varied markedly, depending upon which SNP-haplotype carried the HLA-haplotype. Such an observation indicates that the observed disease-associations were not due to these specific HLA alleles but, rather, to something else, which was present on these SNP-haplotypes (see text). For unclear reasons, this data set did not replicate the findings of Chao and coworkers [19] with respect to the HLA-B*08, HLA-B*13, HLA-B*27, HLA-B*32, and HLA-B*52 haplotypes (see text). In the WTCCC data, however, vast majority (96−100%) of the haplotypes that carried these *HLA-B* alleles, when they included the HLA-DRB1*15:*01* allele, also carried the (*a1*) SNP haplotype. As a result, because they also carried the (*a1*) SNP haplotype, each of these haplotypes was strongly associated with an increased MS-risk except for the extremely rare HLA-B*52~HLA-DRB1*15:*01~a1* haplotype (where OR = 1.01).

Despite the extremely strong association of the (*a1*) SNP-haplotype with this particular HLA haplotype, some *HLA*-DRB1*15:*01~HLA*-DQB1*06:*02* motifs occur in association with other SNP-haplotypes and some of these combinations seem not to have any disease-association (Fig 5A). Thus, for example, single-copy carriers of either *HLA*-DRB1*15:*01~HLA*-DQB1*06:*02~a27* or the *HLA*-DRB1*15:*01~HLA*-DQB1*06:*02~a36* haplotypes, seem not to have any increase in their disease-risk (OR = 0.7; CI = 0.3−1.3 and OR = 0.7; CI = 0.2−2.2, respectively). These ORs are significantly different for both the (*a27*)-containing haplotype (z = 2.5; p = 0.01) and for the (*a36*)-containing haplotype (z = 4.2; p<10^−4^) compared to the same HLA-haplotype containing (*a1*). Similarly, as shown in Fig 5A, considering together all non-(*a1*)-containing haplotypes carrying the *HLA*-DRB1*15:*01~HLA*-DQB1*06:*02* motif these also had significantly smaller ORs than the (*a1*)-containing haplotypes (z = 3.9; p<10^−4^). By contrast, single copy carriers of the (*a1*) SNP haplotype who lack the *HLA*-DRB1*15:*01~HLA*-DQB1*06:*02* HLA haplotype, still have a significantly increased disease risk (OR = 2.2; CI = 1.2–3.8). Moreover, although this OR is less than that found for single copy carriers of the *HLA*-DRB1*15:*01~HLA*-DQB1*06:*02~a1* haplotype, the confidence intervals overlap and the two ORs did not differ significantly (z = 1.2; p = 0.24).

**Fig 6:**
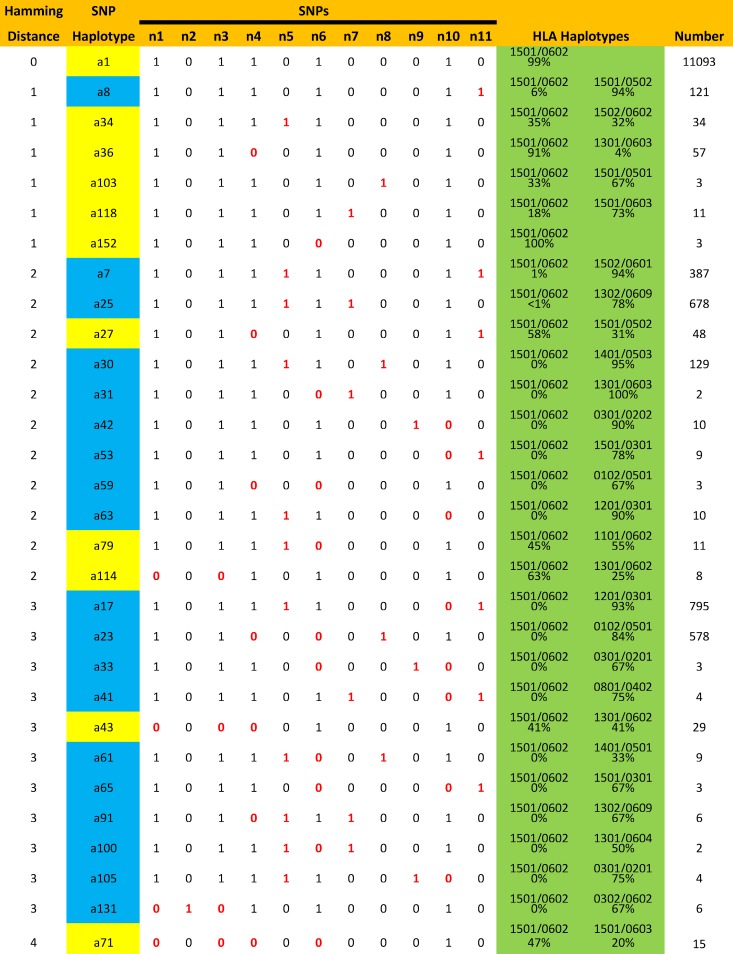
Different SNP haplotypes at distances of 1 to 4 hamming units from the *a1* SNP haplotype (SNP differences highlighted in red; for SNP definitions see text). Several of these SNP haplotypes (indicated in yellow), at times, carried the *HLA*-DRB1*15:*01~HLA*-DQB1*06:*02* HLA haplotype whereas others (indicated in blue) never did. HLA haplotypes are highlighted in green. Thus, whether or not a given SNP haplotype carried this HLA haplotype seemed to be, not a function of the hamming distance, but rather, a property of the specific SNP haplotype involved.

**Fig 7:**
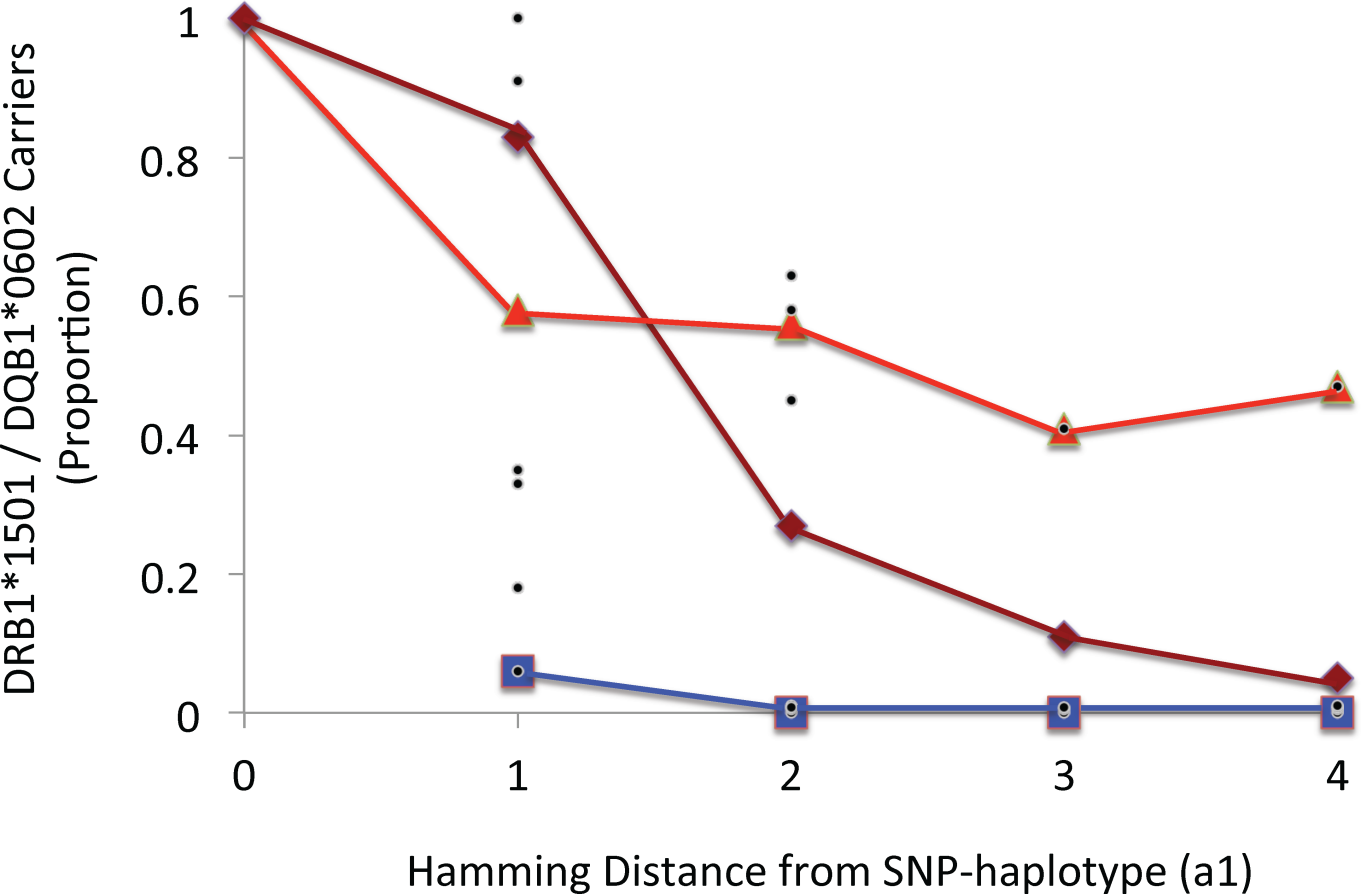
Plot of the proportion of carriers of the *HLA*-DRB1*15:*01~HLA*-DQB1*06:*02* haplotype at different hamming distances from the (*a1*) SNP haplotype. The magenta line represents the average of all haplotypes at a given Hamming distance. Also plotted are the subgroups of haplotypes carrying *HLA*-DRB1*15:*01~HLA*-DQB1*06:*02* less than 10 percent of the time (blue) and those carrying this HLA haplotype 10 or more percent of the time (orange line). Black dots represent individual observations. Certainly, as hamming distance increased, the percentage of haplotypes carrying *HLA*-DRB1*15:*01~HLA*-DQB1*06:*02* diminishes (magenta). However, even at a hamming distance of 4, some specific SNP haplotypes carry this HLA haplotype almost half of the time.

In the WTCCC dataset, the HLA alleles were imputed [44] and, thus, it is possible that either errors of imputation or errors in SNP identification could have influenced these findings. We addressed these possibilities in two ways. First, we compared the HLA associations of the different SNP haplotypes in the imputed WTCCC dataset with the HLA haplotype associations in the Expression, Proteomics, Imaging, and Clinical (EPIC) Study dataset, which had been determined by sequence based typing methods [30]. There was an excellent agreement in the corresponding Class II SNP haplotype associations found in the two datasets (Table 1). In addition, several of the rare *HLA*-DRB1*15:*01~HLA*-DQB1*06:*02* containing SNP haplotypes were found in both datasets (Table 1). Second, we analyzed the hamming distance between the various *HLA*-DRB1*15:*01~HLA*-DQB1*06:*02* containing SNP haplotypes to assess how close these haplotypes were to each other (Figs 6 and 7). Presumably, if errors in SNP identification were responsible for occasionally assigning the *HLA*-DRB1*15:*01~ HLA*-DQB1*06:*02* haplotype to rare SNP haplotypes, the percentage of these errors would tend to be higher for haplotypes at short hamming distances from (*a1*). However, no such relationship was evident (Figs 6 and 7).

#### HLA-DRB1*03:01~HLA-DQB1*02:01

The haplotype *HLA*-DRB1*03:*01~HLA*-DQB1*02:*01* is divided between the (*a2*) and the *a6* SNP haplotypes (Figs 2 & 5B; Table 3). These two haplotypes seem to have distinctive disease associations. Thus, the *a2*-containing haplotype show dominance (or dose dependence), such that both the heterozygotes and homozygotes have an increased disease risk (Fig 5B). This is the case for all the common *a2*-containing extended haplotypes (Table 3). By contrast, the (*a6*)-containing haplotypes, for the most part, show a recessive pattern such that heterozygotes seem not to have any increased risk (Fig 5B). Thus, the increased risk in (*a2*)-containing heterozygotes is significantly different from the (*a6*)-containing heterozygotes (z = 5.9; p<10^−8^), and, in addition, the (*a6*)*-*containing homozygotes have a substantially increased disease risk, which is significantly greater than that found for *a6*-containing heterozygotes (z = 8.0; p <10^−14^). Again, the lack of any increased risk for heterozygotes seems to be true for most of the (*a6*)-containing CEHs (Table 3). However, this was not the case for the extended haplotype *HLA*-A*24:*02~HLA*-C*07:*01~HLA*-B*08:*01~HLA*-DRB1*03:*01~HLA*-DQB1*02:*01~a6*. Thus, for this haplotype, the disease risk for the heterozygote was both significantly increased (Table 3) and, with the exception of (*c27*) and (*c90*), significantly greater than that for other (*a6*)-containing CEHs (range of z-scores: 2.2–4.6; range of p-values: 0.03–10^−5^).

**Table 3:** Common *a2-*, *a6-*, or *a14-*containing (or other) extended haplotypes^††^.

	HLA Haplotype						
Name^†^	*A~C~B~DRB1~DQB1~SNP*		Frequency		OR*		p-value**
*c23*	*30*:*02~05*:*01~18*:*01~03*:*01~02*:*01~a2*		212		2.0 (1.4–2.7)		< E-4
*c46*	*01*:*01~07*:*01~08*:*01~03*:*01~02*:*01~a2*		128		2.1 (1.5–3.0)		< E-4
*c85*	*02*:*01~05*:*01~18*:*01~03*:*01~02*:*01~a2*		75		1.7 (1.0–2.9)		< 0.05
*c1*^*§*^	*01*:*01~07*:*01~08*:*01~03*:*01~02*:*01~a6*		3782		1.1 (1.0–1.2)		< 0.05
*c14*	*02*:*01~07*:*01~08*:*01~03*:*01~02*:*01~a6*		397		0.9 (0.7–1.2)		ns
*c27*	*03*:*01~07*:*01~08*:*01~03*:*01~02*:*01~a6*		181		1.7 (1.2–2.3)		< E-2
*c51*	*68*:*01~07*:*01~08*:*01~03*:*01~02*:*01~a6*		121		0.6 (0.4–1.0)		< 0.05
*c68*	*24*:*02~07*:*01~08*:*01~03*:*01~02*:*01~a6*		91		3.0 (1.8–4.9)		< E-5
*c90*	*03*:*01~07*:*02~07*:*02~03*:*01~02*:*01~a6*		71		1.6 (0.9–2.6)		ns
*c97*	*32*:*01~07*:*01~08*:*01~03*:*01~02*:*01~a6*		68		1.1 (0.6–2.0)		ns
*c110*	*25*:*01~07*:*01~08*:*01~03*:*01~02*:*01~a6*		63		1.3 (0.7–2.3)		ns
*c34*	*68*:*02~08*:*02~14*:*02~13*:*03~03*:*01~a14*		161		1.9 (1.3–2.8)		< E-3
*c96*	*66*:*01~17*:*01~41*:*02~13*:*03~03*:*01~a14*		69		2.6 (1.5–4.5)		< E-3
*c107*	*02*:*01~17*:*01~41*:*02~13*:*03~03*:*01~a14*		64		1.9 (1.1–3.4)		< 0.05
*c5*^*§§*^	*02*:*01~05*:*01~44*:*02~04*:*01~03*:*01~a3*		906		0.5 (0.4–0.6)		< E-11
*c15*	*02*:*01~06*:*02~13*:*02~07*:*01~02*:*02~a3*		361		0.5 (0.3–0.6)		< E-5
*c18*	*02*:*01~06*:*02~57*:*01~07*:*01~03*:*03~a5*		293		0.5 (0.3–0.7)		< E-4
*c24*	*02*:*01~01*:*02~27*:*05~01*:*01~05*:*01~a9*		211		0.5 (0.3–0.7)		< E-3
*c30*	*02*:*01~05*:*01~44*:*02~11*:*01~03*:*01~a4*		173		0.6 (0.4–0.9)		< 0.05
*c32*	*03*:*01~07*:*02~07*:*02~13*:*01~06*:*03~a18*		166		0.6 (0.4–0.9)		< E-2
*c73*	*02*:*01~15*:*02~51*:*01~09*:*01~03*:*03~a4*		87		0.4 (0.2–0.8)		< E-2
*c81*	*24*:*02~07*:*02~39*:*06~08*:*01~04*:*02~a16*		79		3.1 (1.8–5.5)		< E-4

†† haplotypes with ≥ 50 representations in the WTCCC. All such haplotypes carrying the *a2*, *a6*, or *a14* SNP haplotype are included. For each of the listed haplotypes, the Class I and Class II portions were significantly associated with each other far beyond the Bonferroni-adjusted level of significance.

† Arbitrary name for haplotype (sorted in descending order of frequency) for the entire WTCCC population.

* Odds ratio (OR) of disease for individuals having 1 copy of the listed haplotype compared to having no copies of the particular *HLA*-*DRB1~HLA*-*DQB1~SNP* Class II haplotype (95% CI range in parenthesis). All haplotypes carrying the *HLA*-DRB1*15:*01~HLA*-DQB1*06:*02~a1* Class II motif were excluded in this analysis. A Bonferroni correction for the number of haplotypes with 50 or more representations (146) would require a significance level of (p<3*E-4).

** Significance of the association between having 1 copy of the specific allele and the disease (MS) compared to having no copies. The p-values are expressed in scientific notation as powers of 10 (E); ns = not significant. With exception of *c23* and *c46*, all observations with *p<0*.*001* still showed a statistically significant effect even after adjustment for population stratification, geographic, stratification, and gender. Moreover, even *c23* and *c46* trended in this direction (*p≈0*.*10*)

§ Only the *c1* haplotype had enough observations to explore the disease association for having two copies of an allele compared to having no copies of the *HLA*-DRB1*03:*01~HLA*-DQB1*02:*01~a6* Class II haplotype. Thus, this OR was

For *c1*: OR [two copies] = 2.1 (1.5–2.9); *p =* 2.1*E-6

This effect was still statistically significant even after adjustment for population stratification (*p =* 3.13*E-6).

The other Class II haplotypes containing *HLA*-DRB1*03:*01~HLA*-DQB1*02:*01~a6*, combined, had an OR of:

OR [two copies] = 0.8 (0.1–3.4); *p =* ns

§§ This group of haplotypes is composed of those that also had a significant association with this disease. Most of these haplotypes seem to be protective and this protective effect remained significant (p<0.05) even after excluding all individuals who carried the *HLA*-DRB1*15:*01~HLA*-DQB1*06:*02~a1* haplotype.

#### HLA-DRB1*13:03~HLA-DQB1*03:01

The haplotype *HLA*-DRB1*13:*03~HLA*-DQB1*03:*01* is essentially confined to the (*a14I)* SNP haplotype (Figs 2 & 5B; Table 3). This haplotype was clearly associated with an increased disease risk in the heterozygote (Fig 5B); roughly similar for all the most common (*a14*)-containing extended haplotypes (Fig 5). The disease risk may also be increased in individuals homozygous for this haplotype although there were too few observations to be sure (Fig 5B).

#### Other extended haplotypes

Several other CEHs also seemed to be associated with disease risk (Table 3). Many of these were protective and this protective effect was evident despite the fact that those individuals who carried the *HLA*-DRB1*15:*01~HLA*-DQB1*06:*02~a1* haplotype were removed from the analysis (Table 3). By contrast, as is also shown in Table 3, the extended haplotype *HLA*-A*24:*02~HLA*-C*07:*02~HLA*-B*39:*06~HLA*-DRB1*08:*01~HLA*-DQB1*04:*02~a16* was associated with a significant increase in disease risk (OR = 3.0; CI = 1.8–5.5).

Regression analysis confirmed the significance of these observations and no significant interactions were identified. Moreover, adjustment for population stratification, geographic stratification and for gender did not alter these findings (Tables 2 and 3).

#### The EPIC cohort

The cohort of patients from the EPIC study was considerably smaller than those in the WTCCC study and, consequently, only a limited amount of comparative information is available. For example, only 6 CEHs (*c1*, *c2*, *c3*, *c5*, *c6*, *and c11*) had 20 or more representations in the EPIC dataset (*S3 Table*). Nevertheless, all four of the *HLA*-DRB1*15:*01~ HLA*-DQB1*06:*02~a1* containing haplotypes (*c2*, *c3*, *c6*, *and c11*) were significantly associated with MS and had ORs [single copy] ranging from 2.5 to 3.9, with the largest being for (*c11*) and the smallest being for (*c2*). The haplotype (*c1*) had a non-significant OR [single copy] of 1.3 and the haplotype (*c5*) had an OR, which was significantly less than one (OR [single copy] = 0.2). In general these findings are consistent with those reported above for the WTCCC cohort (Tables 2 and 3; *S3 Table*).

## Discussion

In the WTCCC dataset, the MHC region seemed to be composed, largely, of a relatively small collection of very highly-selected CEHs (see *S1 File*) stretching, at least, from the *HLA*-*A* locus to beyond the *HLA*-*DQB1* locus (a distance spanning more than 2.7 mb of DNA). The occurrence of homozygous CEHs was increased both in cases and controls. Such an increase might be expected in the patient population, where the homozygotes of certain haplotypes have an especially high disease risk [9,13–20]. However, it should not be the case for the control population if a balancing selection (i.e., one in which some heterozygous combinations have higher fitness than homozygous combinations) was expected [41]. Alternatively, such a finding might be due to population stratification effects. Thus, such an increase might be expected if local sub-populations had different CEH frequencies (e.g., like Fig 4, but with finer grained population subdivisions) and if individuals from these sub-populations had a propensity to find mates within their same sub-population [45].

Also, and as developed more fully in *S2 File*, when classifying the WTCCC haplotypes into “*rare*” and “*frequent*” CEHs (i.e., those found once or more than once, respectively), there is a significant excess in the number of homozygotes observed for both “*rare*” and “*frequent*” CEHs compared to HWE expectations. For this analysis, homozygotes are considered “*rare−rare*” and “*frequent−frequent*” individuals regardless of the actual CEHs that make up the haplotype pair. The conversion of CEHs from “*rare*” to “*frequent*” or *vice versa* can be caused either by biologic mechanisms (e.g., recombination or mutation) or by mistakes (e.g., typing, imputation, or phasing errors). These errors cannot be avoided entirely due to the marked similarity of many HLA alleles [46]. However, regardless of the underlying mechanism, haplotype conversion, by itself, does not produce any deviation from HWE (*S2 File*). Also, mistakes don’t produce actual changes in CEH frequencies that accumulate over time. By contrast, over time, actual haplotype conversions (e.g. those caused by biologic mechanisms), which are unopposed, would reach a stable state in the population only once the net conversion rate is zero–i.e., when the probability of *frequent→rare* and *rare→frequent* transitions are equal (*S2 File*). This, however, is decidedly not the state of the WTCCC, the EPIC, or other populations here, each of which is composed predominantly of a small number of very common CEHs (Fig 3; *Figure B in S3 File*). Consequently, it must be that the force of actual haplotype conversion is being opposed by another force (i.e., selection) that both retains “*frequent*” CEHs in the population and also perturbs HWE (*S2 File*). Such a selection is already strongly suggested just based on the typical CEH composition of the different human populations (Fig 3, *Figure B in S3 File*). Indeed, using the observed magnitude of the deviation from HWE, and presuming the forces of selection and haplotype conversion balance each other, leads both to the conclusion that the relative probability of survival for individuals with homozygous “*rare*” CEHs is less than 80% of that for individuals with homozygous “*frequent*” CEHs and also that the net *frequent → rare* haplotype conversion rate is on the order of 3−6% for the MHC region in each generation (*S2 File*).

Naturally, there are possible explanations, other than selection, which could also produce a deviation from HWE expectations. Most conspicuous and widely recognized among these is the possibility that the WTCCC population is composed of two or more sub-populations, each of which is in HWE but with each sub-population having different haplotype frequencies. Such a circumstance would violate the HWE assumption of random mating and would lead to the circumstance in which homozygotes are in excess of expectations (as we observed). Moreover, there is no doubt that the exact CEH composition of the WTCCC varies considerably from region to region (e.g., Fig 4; *S2 Table*). Nevertheless, as discussed in *S2 File*, there are several reasons that even this simple mechanism seems inadequate to account for our observed deviations from HWE, Most importantly, we examined the impact that the observed differences in the percentage of “*rare*” CEHs among the sub-populations would have had on the HWE deviation. This analysis indicated that these differences could account for only about a quarter of the difference in HWE that we actually observed (*S2 File*). Consequently, our observations seem likely to be the result of a combination of both haplotype conversion and haplotype selection–each representing processes that take place in every generation.

Moreover, the strong selection of CEHs implies that certain allelic combinations “work well together” whereas other combinations do not (*S2 File*). Presumably, this “working well together”, in a biological sense, means that a particular combination of these five alleles (but likely also including other specific alleles of the many intervening genes) permit the host to respond to a variety of abiotic and biotic threats (or opportunities) in a manner that improves fitness (regardless of whether these come from the external environment, the internal microbiome, or both). However, it is also clear from these findings that no single allelic combination is being selected above all others. Rather, a relatively small number (in the hundreds) of combinations are being selected simultaneously (e.g., Tables 2 and 3; *S1 Table*). Perhaps this is because the nature of these abiotic and biotic threats (or opportunities) result in a very complex “fitness landscape”, which is highly variable both in space and in time and, thus, in which fitness depends upon the precise environmental context of the individual, including specific host factors such as the exact location of their residence, their particular micro-environment, their diet, their lifestyle, or other individual idiosyncrasies. In such a case, no single CEH may be favored in all circumstances and, consequently, such highly variable landscape topography might help to rationalize why so many haplotypes seem to be selected simultaneously. It might also help to rationalize why the group composition of the selected CEHs seems to be so fluid between separated populations (e.g., Fig 4; *S1 Table*). Thus, even within European populations, the beginning of such a divergence can already be recognized (Fig 4; *S2 Table*) and, based on limited data, this divergence in the group composition of the selected haplotypes in long separated populations (i.e., Africans, AmerIndians, Asians, and Caucasians) seems to be substantially greater (*S1 File; S1 Table*).

The main hypothesis of the present study was that any observed allelic disease association is a reflection of those CEHs, which confer MS disease risk. The present study sheds considerable light on this hypothesis. For example, although many CEHs, which include the Class II motif HLA-DRB1*15:01~HLA-DQB1*06:*02~a1*, are associated with an increased disease risk (Table 2), the actual risk varies significantly among the different extended haplotypes (Table 2; *Figure C in S3 File*). Moreover, some haplotypes, which include the motif HLA-DRB1*15:01~HLA-DQB1*06:*02* but don’t include the SNP-haplotype *a1*, seem not to carry any risk (Fig 5A). By contrast, the (*a1*)-containing haplotypes, which don’t include this Class II motif, still carry substantial risk (Fig 5A). These observations suggest that the motif of HLA-DRB1*15:01~HLA-DQB1*06:*02*, by itself, does not fully account for the disease risk associated with these extended haplotypes. Regardless of this conclusion, however, some disease risk seems to be attributable to some aspect of the HLA-DRB1*15:01~HLA-DQB1*06:*02~a1* haplotype by itself. Thus, even correcting for population stratification effects, the disease risk is still significantly increased for those individuals who both carry this Class II haplotype and, yet, whose full extended haplotype had only a single representation in the WTCCC.

In the case of the Class II HLA motif of HLA-DRB1*03:01~HLA-DQB1*02:*01*, this dependence on the extended haplotype is even more evident. Thus, most of the common extended haplotypes, which include the Class II motif of HLA-DRB1*03:01~HLA-DQB1*02:*01~a2* seem to associate with a disease risk that is either dominant or dose dependent (Table 3; Fig 5B). By contrast, those haplotypes, which include the motif of HLA-DRB1*03:01~HLA-DQB1*02:*01~a6*, as a group, seem to associate with a disease risk that is recessive (Fig 5B). Nevertheless, at least one of these (*a6*)*-*containing haplotypes (i.e., HLA-A*24:02~C*07:01~HLA-B*08:01~HLA-DRB1*03:01~HLA-DQB1*02:*01~a6*) is associated with a disease risk, which is either dominant or dose dependent (Table 3).

In summary, the MHC is organized into a relatively small group of extended haplotypes (CEHs), which seem to be under a very strong selection pressure, presumably based upon favorable biological properties of the complete haplotype. If so, then, of necessity, this means that disease susceptibility is probably not attributable to any specific HLA allele but rather susceptibility is likely to be dependent upon the nature of each CEH. This conclusion seems to be borne out by the data. Moreover, it is of note that the most highly selected of these CEHs (in Caucasians) also seem to be the ones most likely to be associated with and increased risk of MS. The reasons for this apparent relationship are unclear. However, it is a fact that for the WTCCC population as a whole, for each of the WTCCC regions individually (Fig 4), and also for the EPIC cohort, the three most common CEHs (and 11 of the most common 25 CEHs) were associated with a significantly increased risk of MS (Tables 2 and 3; *S3 Table*). This observation that the most highly-selected CEHs also carry the greatest MS risk presumably indicates that there must be a net survival advantage for individuals carrying these CEHs, which outweighs the small increased chance of getting MS–a circumstance that is also suggested by the observation (*Figure A in S3 File*) that only a very small proportion of the individuals who carry these disease-associated CEHs are even within the set of individuals who are “genetically susceptible” to getting the disease [3].

## Materials & methods

### Ethics statement

This research has been approved by the University of California, San Francisco's Institutional Review Board (IRB) has been conducted according to the principles expressed in the Declaration of Helsinki.

### Study participants

#### Wellcome Trust Case Control Consortium (WTCCC)

The study cohort was assembled as a prospective multicenter, multinational, effort. This study population has been described in detail previously [12,14, 16, 17]. However, in brief, this cohort included 18,872 controls and 11,376 cases with MS, although SNP haplotype data was unavailable for 380 controls and 232 cases. Of the cases, 72.9% were women, the average age-of-clinical-onset was 33.1 years, and the mean Extended Disability Status Score (EDSS) was 3.7 [12]. Fifteen different countries from around the world participated (Australia, Belgium, Denmark, Finland, France, Germany, Ireland, Italy, Poland, New Zealand, Norway, Spain, Sweden, the United Kingdom, and the United States). The data from Australia and New Zealand were combined so that data from 14 different world regions was available. Consequently, the patients enrolled in this study (except for a few African Americans from the United States) were of European ancestry. Although all clinical MS subtypes were included, the large majority (89%) had a relapsing-remitting onset [11]. The diagnosis of MS was made based upon internationally recognized criteria [47–49]. Control subjects were composed of a combined group, which consisted of several different cohorts of healthy individuals with European ancestry [11]. The Ethical Committees or Institutional Review Boards at each of the participating centers approved the protocol and informed consent was obtained from each study participant. The WTCCC granted data access for this study.

#### Expression, Proteomics, Imaging, and Clinical (EPIC) study

An independent cohort, for certain comparative purposes, consisted of the patients and controls enrolled in the EPIC study of MS genetics at UCSF and this cohort, also, has been described in detail previously [8]. Briefly, this study included data from 964 patients with MS and 868 controls. Both patients and controls were matched for age and gender, and all participants provided their informed consent [8]. The cohort was drawn from the United States and, essentially, all participants were of European ancestry. The diagnosis of MS, also, was made using internationally recognized criteria [47–49].

### Genotyping, and quality control

The genotyping methods and quality control for the WTCCC have been described in detail previously [11,12]. All genotyping was performed on the Illumina Infinium platform at the Wellcome Trust Sanger Institute. Case samples were genotyped using a customized Human660-Quad chip. Common controls were genotyped on a second customized Human1M-Duo chip (utilizing the same probes). After quality control, this provided data on 441,547 autosomal SNPs scattered throughout the genome in both MS patients and controls [17]. The identities of the five HLA alleles in the MHC region (*A*, *C*, *B*, *DRB1* and *DQB1*) were determined for each participant by imputation using the HIBAG method [44].

Genotyping and quality control methods for the EPIC cohort have also been described in detail previously [7]. In this study, SNP genotyping was done at the Illumina facilities using the Sentrix HumanHap550 Bead Chip. This analysis provided genotype information on 551,642 SNPs. The identities of the five HLA alleles in the MHC region (*HLA-A*, *HLA-C*, *HLA-B*, *HLA-DRB1* and *HLA-DQB1*) were determined by sequence based typing methods [28].

### Statistical methods

#### Phasing

The phasing of alleles at each of five HLA loci (*HLA-A*, *HLA-C*, *HLA-B*, *HLA-DRB1* and *HLA-DQB1*) was accomplished using a previously published probabilistic phasing algorithm [50, 51]. Phased SNP haplotypes were constructed using a previously published probabilistic method [29, 30] at sliding windows of 2 to 15 SNPs throughout the 1 mb span surrounding the Class II region of the *DRB1* gene. The SNP-window of the most significant MS-associated SNP haplotype was carried forward as a haplotype locus, a multi-allelic gene to be phased with the 5 classic HLA genes. As discussed earlier, this haplotype locus consisted of 11 phased SNPs surrounding the *HLA-DRB1* gene (Fig 1). The accuracy of the phasing was confirmed by the method of SHAPEIT2 [52–54], with better than 99% correspondence between methods.

Phasing was accomplished by determining the probability of each possible combination and assigning the phasing to the most likely combination. At times, however, there were several possible combinations and this method, potentially, might designate a haplotype pair in circumstances where several compatible haplotype pairs existed and each pair had a very similar posterior probability. Such a situation did occur, but rarely. Thus, for the *HLA-A~HLA-C ~HLA-B~HLA-DRB1~HLA-DQB1* haplotypes, 98% of the designations had a posterior probability of more than (0.5), 92% had posterior probability of more than (0.6), and 85% had a posterior probability of more than (0.7). For the Class II haplotypes (*HLA-DRB1*~*HLA-DQB1~SNP*), these same respective percentages were (100%, 99.997%, and 99.95%).

#### Haplotype frequencies and association testing

Disease association tests, as measured by ORs and confidence intervals (CIs), were undertaken for each of the HLA haplotypes and HLA plus SNP haplotypes. Because of the very strong association between the *HLA*-DRB1*15:*01~HLA*-DQB1*06:*02~a1* haplotype, all other associations were assessed after excluding those individuals who carried the *HLA*-DRB1*15:*01~HLA*-DQB1*06:*02~a1* haplotype. Similarly, when the association of a specific CEH carrying the *HLA*-DRB1*15:*01~HLA*-DQB1*06:*02~a1* haplotype was assessed, all other *HLA*-DRB1*15:*01~HLA*-DQB1*06:*02~a1* carriers were excluded from the analysis.

In our previous study [30] we found an association of certain Class I alleles with MS (i.e., HLA-A*02:*01*, HLA-C*05:*01*, HLA-B*37:*01*, HLA-B*38:*01*, and HLA-B*44:*02*). Consequently, for each of the reported Class II associations (Fig 5), we undertook a regression analysis using these Class I alleles as covariates in the regression equations. This analysis confirmed that the reported Class II associations (Fig 5) were unaffected.

In our previous report [30] we assessed the significance of the association of each SNP haplotype with MS and adjusted these associations for the millions of comparisons made across the entire genome using the Benjamini-Hochberg method [55]. In the present manuscript, by contrast, we analyzed the 174 distinct SNP haplotypes composed of variants at 11 SNP locations *(rs2395173*, *rs2395174*, *rs3129871*, *rs7192*, *rs3129890*, *rs9268832*, *rs532098*, *rs17533090*, *rs2187668*, *rs1063355*, *and rs9275141*). Among these haplotypes was the (*a1*) SNP-haplotype (Table 1), which had the single largest disease-association with MS of any in the genome. In the present manuscript, however, these 174 SNP haplotypes in this genomic region served simply (and only) as an additional genetic marker to be included in the haplotype analysis with the other 5 HLA loci and, thus, no additional statistical adjustment is necessary (or appropriate) as a consequence of their inclusion in the analysis. Nevertheless, because only haplotypes with 50 or more representations in the WTCCC dataset were analyzed, and because there were 146 such haplotypes, a Bonferonni correction for these multiple comparisons would require a significance of (p < 0.05/146 = 0.0003) to be achieved.

Because of the tight linkage that exists among the Class II loci (*HLA-DRB1*, *HLA-DQB1*, and *SNP* haplotype) as well as among the Class I loci (*HLA-A*, *HLA-C*, and *HLA-B*), the association of the different Class I and Class II haplotype combinations (with more than 2 representations in the WTCCC dataset) was determined by the association of specific *HLA-A*~*HLA-C*~*HLA-B* combinations with a specific *HLA-DRB1~HLA-DQB1~SNP* haplotype combinations. The *p*-values for the association of different Class I with different Class II combinations were determined using a Fisher exact test if any expected cell frequencies was 5 or less and otherwise using a Chi square test [56]. The Benjamini-Hochberg method was used to correct for multiple testing of the different possible Class I / Class II combinations [55].

Significance of the difference in ORs for disease association between any two haplotypes was determined by z-scores calculated from the difference in the natural logarithm of the ORs for the haplotypes. Also, because of the marked predominance of the MS association with the HLA-DRB1*15:01~HLA-DQB1*06:*02~a1* haplotype, all disease association tests for other haplotypes were assessed after persons carrying the HLA-DRB1*15:01~HLA-DQB1*06:*02~a1* haplotype were excluded from the analysis. Similarly, in the case of disease association tests for individual CEHs that carried the HLA-DRB1*15:01~HLA-DQB1*06:*02~a1* Class II motif, all other persons carrying this same Class II motif were excluded from the analysis.

Significance of disease associations were also confirmed using a regression analysis equating phenotype (case or control) with the dose (0, 1, or 2) of each of haplotypes identified as being disease associated. An analysis of the potential interactions between the haplotypes was also undertaken with these regression equations.

The expected occurrence of individuals homozygous for the different CEHs (or different CEH-types) was calculated from the measured CEH (or CEH-type) frequencies. These individual expectations were then summed and the expected total compared to the observed total number of homozygous individuals using a z-score.

#### Population stratification

We used principal components (PC) analysis excluding MHC SNPs (Eigensoft) to correct for population stratification within the WTCCC cohort [57]. There was evidence of considerable population structure in the WTCCC data. An analysis of variance test carried out between cases and controls demonstrated a significant difference for most of the first 10 PCs (which accounted for 84% of the of the population stratification). None of other PCs were significantly different between cases and controls (neither were PC4 or PC10). The potential impact of this population structure on our findings was assessed by the inclusion of these 10 PCs in the final regression equation.

#### Geographic, gender, and age stratification

We also adjusted for geographic heterogeneity (in addition to our adjustment for population stratification) by using dummy variable coding for each of the different geographic regions and including these in the final regression equation. Similarly, and adjustment for gender (male = 1; female = 0) was also included in the final regression equation. Neither information about the individual chronological age nor information about individual age-at-clinical-onset was available for either the WTCCC of EPIC data sets. Nevertheless, because this study analyzed only DNA-based haplotypes (which are independent of chronological age), chronological age is not a relevant factor. It is possible, however, that the age at disease-onset could be more relevant. Certainly, some authors have argued that “childhood-onset” MS cases might somehow be different (either genetically or environmentally, or both) from “adult-onset” cases. Nevertheless, within an “adult-onset” MS population (e.g., the WTCCC population), there is no evidence to suggest genetic heterogeneity with respect to age-at-clinical-onset. Also, it is worth pointing out that many patients with “adult-onset” MS, can be demonstrated to have MRI evidence of disease activity that precedes, by many years (oftentimes decades), the clinical-onset of MS. Moreover, there is no established (or suggested) relationship between the age-at-clinical-onset and the age of disease-onset. Consequently, any analysis, regarding the impact of the age at disease-onset based solely upon the age observed at the clinical-onset of disease activity, would be unreliable, even if such data were available.

## Supporting information

S1 File This section considers the wide-spread occurrence of high frequency CEHs in different human populations and also how the WTCCC population differs from certain other populations of the world. (PDF)

S2 File This section develops the mathematical model for understanding the dynamics of haplotype conversion and selection as they relate to the MHC. This is the model used in the Text to estimate the values of these two parameters from the WTCCC and EPIC data.
(PDF)

S3 File This section includes the data for *Figures (A–C)*. (PDF)

S1 Table Conserved extended haplotypes (CEHs) in the WTCCC, the EPIC, and in other populations. This includes an estimate of the overlap (in % of the total number of CEHs) of each ethnicity with the CEHs from another ethnicity.
(XLSX)

S2 Table Variations in the frequency of the 25 most common CEHs in different geographic regions participating in the WTCCC and in the EPIC. (XLSX)

S3 Table Common extended haplotypes (CEHs) in the EPIC population. (DOCX)
