# Supplementary material for: Highly conserved extended haplotypes of the major histocompatibility complex and their relationship to multiple sclerosis susceptibility
Source: PLoS One. 2018 Feb 13;13(2):e0190043. doi: 10.1371/journal.pone.0190043 (PMC5810982; doi:10.1371/journal.pone.0190043)
Supplement: S1 File — (PDF) [file pone.0190043.s001.pdf]

Supplemental Material #1

Highly Conserved Haplotypes of the MHC

Several highly conserved extended haplotypes (CEHs) in the MHC region are known to involve both Class I (*HLA-A~HLA-C~HLA-B*) and Class II (*HLA-DRB1~HLA-DQB1~SNP*) loci – a region which spans a genomic distance of more than 2.7 mb of DNA [23-34]. Considering the huge number of possible combinations of alleles at these loci, as discussed in the main text, it is notable that only a relatively small number of very high-frequency haplotypes account for the vast majority of combinations present in the population (*e.g., Supplemental S1 Table*). Moreover, this same imbalance seems to characterize a number of widely divergent populations (*Supplemental Figure B in File S3*). In such circumstances, it is not surprising to find that Class I (*HLA-A~HLA-C~HLA-B*) and Class II (*HLA-DRB1~HLA-DQB1~SNP*) portions of these haplotypes are very strongly associated with each other.

For example, the ten *HLA-A~HLA-C~HLA-B* motifs of the 10 most common CEHs (*Supplemental S1 Table*) were associated with 592 unique extended haplotypes, of which 45% had only a single representation in the WTCCC dataset. Similarly, the 7 *HLA-DRB1~HLA-DQB1~SNP* motifs of the 10 most common CEHs (*see Supplemental S1 Table*) were associated with 2,640 unique extended haplotypes, of which 46% had only a single representation in the WTCCC dataset. The disparity in the number of associated unique haplotypes between the Class I and Class II motifs reflects the greater variability of the (*HLA-A~HLA-C~HLA-B*) Class I region, perhaps due, in part or in whole, to its greater genomic extent compared to the Class II region. Moreover, considering only the CEHs, which had a single representation in the WTCCC, 68% of their *HLA-A~HLA-C~HLA-B* motifs, 87% of their *HLA-DRB1~HLA-DQB1~SNP* motifs, and 40% of their *HLA-C~HLA-B~HLA-DRB1~HLA-DQB1~SNP* motifs were found as part of the more common CEHs in the WTCCC population. Both of these sets of observations point to a considerable degree of mixing of these tightly coupled motifs among the CEHs of the WTCCC, as might be expected from recombination events.

Moreover, for each of the most common 25 haplotypes, the association of the Class I component with the Class II component of the haplotype was markedly greater than chance (range of ORs = 4−122). Thus, 19 of them had a significance of association of ($p<10^{-300}$); 4 had a significance of ($p<10^{-100}$); and 2 had a significance of ($p<10^{-19}$). Also, of the 269 unique haplotypes with more than 30 representations, the significance of the association of the Class I

(*HLA-A~HLA-C~HLA-B*) portion with the Class II (*HLA-DRB1~HLA-DQB1~SNP*) portion was more significant than the Benjamini-Hochberg adjusted level in 251 (93%) of them. Moreover, it is likely that most (or all) of the remaining 18 haplotypes also had a true association between the Class I and II portions because 15 of them had a nominal level of statistical significance ($p<0.05$) and for all (100%) of these haplotypes the direction favored an association. Similarly, for the 733 unique haplotypes with more than 10 representations, 482 (66%) of them had and association of more than the Benjamini-Hochberg adjusted level, an additional 207 (28%) had a nominal level of significance, and in most (94%) the direction favored an association. Even for the 2,666 haplotypes with 3 or more representations 699 (26%) had an association with a significance beyond the Benjamini-Hochberg adjusted level, an additional 1,974 (67%) had a nominal level of statistical significance and in most (94%) the observed direction favored an association.

Similarly, examination of the association between the isolated *A* allele and the remaining portion of the CEHs (*HLA-C~HLA-B~HLA-DRB1~HLA-DQB1~SNP*), confirmed the importance of the entire haplotype to its specificity. Thus, of the 269 haplotypes with more than 30 representations, 232 (86%) had an association of the *A* allele with the remainder of the haplotype, which was more significant than the Benjamini-Hochberg adjusted level. An additional 29 (11%) had an association with a nominal level of significance ($p<0.05$) and, in all (100%), the observed direction favored an association. Both other analyses of the isolated *A* allele (i.e., for haplotypes with both more than 10 and more than 3 representations), also conformed to those presented above for the Class I – Class II split, indicating that the *A* allele is an integral component of each CEH.

The strength and consistency of these associations indicated that these CEHs are under considerable selection pressure (*see also Supplemental Material #2 in S2 File*). Previous reports have also found CEHs in the MHC of Caucasian populations [23-28, 30, 35]. For example, in the study of Bugawan and coworkers [23], 18 extended "multiple copy" 5-locus haplotypes in Caucasians accounted for 24% of all haplotypes in their population and, of these 18 haplotypes, 12 were among the 25 most common CEHs in the WTCCC dataset (*Supplemental S1 Table*). Of the 14 extended haplotypes in Caucasians described by Ahmad and coworkers [24], 6 among the 25 most common CEHs in the WTCCC data set (Table 2). Of the 5 extended haplotypes reported by Horton and colleagues [26], 3 were among the 25 most common CEHs in the WTCCC. Of the 17 extended haplotypes for the *A*, *B*, and *DRB1* loci in Caucasians described by Wennerström and coworkers [27], 9 were among the 25 most common CEHs in the WTCCC. And finally, in the small Caucasian population from the report by Zúñiga and coworkers [28], three of the 6

haplotypes were among the 25 most common CEHs of the WTCCC (*Supplemental S1 Table*). Taken together, these five reports describe 36 unique extended haplotypes among the various Caucasian populations, of which 22 were among the 25 most common CEHs of the WTCCC.

Moreover, all but six [23, 24, 27, 28] of these 36 Caucasian haplotypes were, at least, represented in the WTCCC. Four of these haplotypes, which were not found in the WTCCC, were likely typing errors because each differs in only a minor way [46] from very common haplotypes in the WTCCC (i.e., *HLA-DQB1*02:01* instead of *HLA-DQB1*02:02*; *HLA-A*29:01* instead of *HLA-A*29:02*; and *HLA-C*03:03* instead of *HLA-C*0304*). The other two also differed in a relatively minor way from very rare haplotypes in the WTCCC. Nevertheless, the imperfect overlap between the Caucasian haplotypes reported in these studies is reflected by the fact that, of the 36 five-locus haplotypes reported in four studies [23, 24, 26, 28], only five unique haplotypes were found in two of the studies, two were found in three studies, and none were found in all four studies.

Similarly, in the independent EPIC dataset, consisting of 1,832 extended Caucasian haplotypes, all of the 25 most common haplotypes in EPIC, and 79 of the 90 haplotypes (88%) with more than one representation, were also found in the WTCCC. Similarly, each of the 25 most common haplotypes in the WTCCC, were also found in the EPIC cohort. Nevertheless, the frequency distribution of the EPIC haplotypes differed somewhat from the WTCCC (Figure 4; *Supplemental S1 Table*). Finally, in the very large dataset of Gragert and coworkers, consisting of over a million European-Caucasian individuals [35], all 25 of these alleles were found within the 100 most common CEHs reported world-wide (*Supplemental S1 Table*).

The degree (to which it exists) of non-overlap between these Caucasian populations is probably a function of the evolutionary distance between the different groups sampled. For example, even within the WTCCC, the prevalence of each of the 25 top CEHs varied considerably by geographical region (*Supplemental S2 Table*).

By contrast, several populations, which are separated from Caucasians by an even greater evolutionary distance, have much less overlap than any of these Caucasian populations. For example, a recent study from Nigeria also reported a high prevalence of CEHs [31]. In this study, 14 extended, 5-locus, West African haplotypes (*HLA-A~HLA-C~HLA-B~HLA-DRB1~HLA-DQB1*) accounted for 16% of the haplotypes present in their population. Also, in an AmerIndian population, a high prevalence of 5-locus extended HLA haplotypes was, again, reported [28]. In

their mixed population (which included AmerIndians, Caucasians, Africans, and persons of undetermined ancestry), 16 AmerIndian haplotypes accounted for 13% of the total haplotypes reported. Presumably, if the different groups had been segregated in this report [30], this percentage would be higher in the AmerIndian population considered alone.

Interestingly, however, there was essentially no overlap between any of these different groups. Common haplotypes in West Africa and AmerIndians were completely distinct from one another [26,30]. Moreover, in the WTCCC dataset, only a single individual carried a single copy of one of the more common West African haplotypes and no one carried any of the AmerIndian extended haplotypes. Also, in an Asian population, Yunis and coworkers described 10 common 5-locus extended HLA-haplotypes [25]. Of these 10 haplotypes, 4 were also found in the WTCCC dataset although the actual degree of overlap was quite modest. Thus, of the 59,884 haplotypes present in the WTCCC dataset, these 4 "Asian" haplotypes (combined) only accounted for only 80 of them (0.1%). In addition, there was not any overlap of these common Asian haplotypes with those from the West African or the AmerIndian populations.

In the very large dataset of Gragert and coworkers [35], their European population CEH distribution was very similar to that found in the WTCCC and EPIC studies (*Supplemental S1 Table*). Also, similar to the above observations suggesting a marked divergence between long separated populations, Gragert and colleagues [35] found that those CEHs having a frequency in the WTCCC ≥ 0.00025, which represented more than 65% of the WTCCC population, accounted for only 6.7% and 8.1% of the total haplotypes in the African and Japanese populations, respectfully (*Supplemental S1 Table*). By contrast and asymmetrically, when considering the CEHs with a haplotype frequencies of 0.00025 or more in the Gragert et al. [35] study for their African and Japanese populations, these common African and Japanese haplotypes accounted for 34.6% and 23.0% of the European haplotypes that they observed (*Legend of: Supplemental S1 Table*). Such asymmetry could be due to a greater influx of African and Japanese genes into the European population compared to the influx of European genes into the native African and Japanese populations.

Also, these same common WTCCC haplotypes accounted for 44.6% of the total AmerIndian haplotypes (*Supplemental S1 Table*). This observation, together with the similarity of individual CEH frequencies in the Caucasian and the AmerIndian populations (self-identified), suggests that their AmerIndian population is more related to Europeans than they are to Asians, possibly due to an asymmetric admixture from the European-American population.
