# Supplementary material for: Highly conserved extended haplotypes of the major histocompatibility complex and their relationship to multiple sclerosis susceptibility
Source: PLoS One. 2018 Feb 13;13(2):e0190043. doi: 10.1371/journal.pone.0190043 (PMC5810982; doi:10.1371/journal.pone.0190043)
Supplement: S2 File — This is the model used in the Text to estimate the values of these two parameters from the WTCCC and EPIC data. (PDF) [file pone.0190043.s002.pdf]

Supplemental Material #2

Haplotype Conversion and Selection

Haplotype Conversion

Let (p) and (q) be the haplotype (CEH) frequencies for a population in HWE such that:

$$p^2 + 2pq + q^2 = 1 \quad ; \qquad \text{or:} \qquad p + q = 1$$

For the purposes of this analysis, haplotypes consisting of a specific SNP-haplotype [29, 30], together with a specific combination of alleles at each of 5 HLA loci (*A*, *C, B, DRB1,* and *DQB1*), will be analyzed by the frequency of their occurrence in the WTCCC. Haplotypes that have only one representation in the WTCCC dataset will be designated as "*rare*" haplotypes with a population frequency of (p). By contrast, haplotypes with more than one representation in the WTCCC will be designated as "*frequent*" haplotypes with a population frequency of (q). In this circumstance, haplotype conversion from "*frequent*" to "*rare*" or *vice versa* might take place by several mechanisms (e.g., recombination, mutation, random typing or imputation errors, etc.). As an example, in the WTCCC, one person's pair of homozygous "*frequent*" HLA haplotypes (*see Supplemental S2 Table*) was:

*A*11:01~C*04:01~B*35:01~DRB1*01:01~DQB1*05:01~a9* *c10*

*A*68:02~C*08:02~B*14:02~DRB1*13:03~DQB1*03:01~a14* *c34*

In this case, if a crossover were to take place between the *B* and the *DRB1* loci, two new HLA haplotypes would be produced, both of which, after the crossover, would be: "*rare*".

*A*11:01~C*04:01~B*35:01~DRB1*13:03~DQB1*03:01~a14* *"rare"*

*A*68:02~C*08:02~B*14:02~DRB1*01:01~DQB1*05:01~a9* *"rare"*

In such a circumstance, it can be said that each of these "*frequent*" haplotypes has been converted into a "*rare*" haplotype by the crossover.

Naturally such haplotype "conversion" can go in either direction although, because there are a vastly greater number of "*rare*" CEH states compared to "*frequent*" CEH states (*see Main Text)*, the likelihood of CEH conversion should be much more likely to go from "*frequent*" to "*rare*" than *vice versa*. Indeed, constructing such crossovers in the gaps between the *A* and *C* loci for all individuals in the WTCCC and (separately) between the *B* and *DRB1* loci – each gap

spanning a genomic distances of approximately 1 mb – the rate of $(frequent \rightarrow rare)$ conversions was (5.4) times the rate of $(rare \rightarrow frequent)$ conversions.

Consequently, we can define the probabilities of haplotype conversion as:

$$\begin{aligned}&P(frequent \rightarrow rare) = v\\&P(rare \rightarrow frequent) = w\\&where: \quad v \approx 5.4w\end{aligned}$$

In this case, assuming HWE in the original population, the probabilities of haplotypes expected in the next generation (after conversion has taken place) will be:

$$p'_{excpected} = p - pw + qv \quad \text{and:} \quad q'_{excpected} = q + pw - qv$$

and, thus, the expected distribution of homozygous and heterozygous genotypes (given that HWE continues in the new population after conversion) will be:

Homozygous (q'): $(q'_{excpected})^2 = \{q + pw - qv\}^2$ (1)

Heterozygous: $2(p'_{excpected})(q'_{excpected}) = 2(q + pw - qv)(p - pw + qv)$

Homozygous (p') $(p'_{excpected})^2 = \{p - pw + qv\}^2$ (2)

By contrast, the observed haplotype frequency of (p') in the next generation (after conversion) will be:

$$p'_{observed} = \text{homozygous frequency} + \{(0.5) * \text{heterozygous frequency}\}$$

Assuming independence of the conversion events, the homozygous (HZ) frequency is:

$$\begin{aligned}HZ &= p^2 - p^2w^2 - 2p^2w(1-w) + 2pqv(1-w) + q^2v^2\\&= p^2 + p^2w^2 + q^2v^2 - 2p^2w + 2pqv - 2pqvw\\&= (p - pw + qv)^2\end{aligned}$$

And the heterozygous (*HTZ*) frequency is:

$$\begin{aligned}HTZ &= 2pq - 2pqv(1-w) - 2pqw(1-v) + 2q^2v(1-v) + 2p^2w(1-w)\\&= 2pq - 2pqv - 2pqw + 4pqvw + 2q^2v - 2q^2v^2 + 2p^2w - 2p^2w^2\\&= 2(p - pw + qv)(q + pw - qv)\end{aligned}$$

Therefore, the observed frequency of (p') is:

$$\begin{aligned}p'_{observed} &= (p - pw + qv)^2 + (p - pw + qv)(q + pw - qv)\\&= (p - pw + qv)(p + q) = p - pw + qv\end{aligned} \quad (3)$$

Similarly: $q'_{observed} = q + pw - qv$ (4)

Thus, if the previous generation is in HWE, then the current generation will also be in HWE, regardless of the mechanism for net haplotype conversion (e.g., recombination, mutation, random typing or imputation errors, phasing errors etc.). By contrast, systematic typing or imputation errors should lead to the consistent misidentification of certain specific alleles but not to a net conversion of "*frequent*" CEHs to "*rare*" CEHs or *vice versa*. However, even if such errors did have this effect, it would not impact HWE status. Nevertheless, the implied meaning of the terms (v) and (w) would be different for different mechanisms. These two rates incorporate both the likelihood that some event occurs as well as the likelihood, if this event occurs, that a haplotype conversion actually takes place. For example, both the mechanisms of crossovers and of phasing errors will, necessarily, impact both of a haplotype-pair and, thus, the combined conversion rates (v and w) will be less than or equal to twice the event rate (i.e., either the crossover or phasing error rates). By contrast, mechanisms such as mutation and random typing or imputation errors will likely affect only one of a haplotype pair, so that the combined rate will be less than or equal to the event-rate.

Similarly, it is important to distinguish between those biological processes (e.g., mutation and crossover), which lead to an actual change in haplotype frequencies and those processes (e.g., typing, imputation, and phasing errors), which represent mistakes and don't lead to any real change. In addition, mutation and recombination occur principally during oögenesis and spermatogenesis, whereas selection presumably occurs mostly after this developmental time point. By contrast, typing, imputation, and phasing errors occur largely after both selection and actual haplotype conversions have already taken place. Moreover, if true haplotype conversions were the only processes taking place, and if they continued indefinitely, the population would reach stability when there was no further net haplotype conversion (i.e., when: $qv = pw$). Given the observed relationship (i.e., $v \approx 5.4w$) for recombination, and presuming the relationship between (v) and (w) didn't change over time, this balance will occur when approximately 85% of the CEHs in the population are "*rare*". Such a balance is far from the status of the WTCCC, the EPIC, and other populations (Figure 3; *Supplemental Figure B in File S3*).

In reality, however, over time, as "*rare*" haplotypes became more common in the population, the ratio of (v/w) would likely increase substantially as a reflection of the vastly greater number of possible "*rare*" combination-states compared to "*frequent*" combination-states (*see Main Text*). Such a change in the ratio (v/w) would mean that, at stability, almost all of the CEHs in the population would be "*rare*". Such a balance is even more remote from the current status of the WTCCC, the EPIC, and other populations (*Supplemental Figure B in File S3*).

Haplotype Selection

There are, however, other forces (e.g., selection) that will serve to keep "*frequent*" CEHs in the population. Therefore, we will consider the circumstance, in which homozygous "*rare*" genotypes are less likely to survive than homozygous "*frequent*" genotypes and, consequently, less likely to be included in the adult populations of the WTCCC or EPIC. Importantly, as demonstrated in Figure 3 & *Supplemental Figure B in File S3*, human populations seem largely to consist of a very small number of very common "*frequent*" CEHs [23-39]. Consequently, because this population-composition is so wide-spread among geographically separated human groups, it seems that such a structure is a stable feature of the human MHC; this despite the fact that different populations differ markedly from each other with respect to their actual CEH composition (Figure 4; *Supplemental S1 Table*). Therefore, for the purposes of this analysis, we will assume that the frequencies of both "*rare*" and "*frequent*" CEHs are, indeed, stable and that the forces of selection and true haplotype conversion exactly balance each other. In this context, selection is not against "*rare*" CEHs *per se*. Rather, selection is envisioned to occur because "*rare*" CEHs have, on average, certain biological properties that make them less fit compared to the average fitness of "*frequent*" CEHs. Because, as mentioned earlier, errors of typing, imputation, or phasing do not lead to actual changes in haplotype frequency, they can't be opposed by selection, and they don't affect HWE status, we will confine our analysis to actual haplotype conversions caused by either mutation or recombination. We also assume that prior to both haplotype conversion and selection, the population is in HWE.

Defining (1.0) to be the relative survival probability for the most-fit homozygote (assumed, initially, to be the "*frequent*" homozygote), we will let $(0 \le s \le 1)$ be the relative survival probability for the least-fit "*rare*" homozygote and we will let $(hs \mid h \ge 0)$ be the relative survival probability for "*rare − frequent*" heterozygotes. We define (c) such that this represents the make-up of the adult population after both haplotype conversion and selection have already taken place.

Thus, we will let:

$$c = (q - qv + pw)^2 + 2hs(q - qv + pw)(p + qv - pw) + s(p + qv - pw)^2 \qquad (5)$$

In this case, the observed frequencies for the homozygous "*rare*" genotype, the heterozygous "*rare – frequent*" genotype, and the "*rare*" haplotype are:

Homozygous: $HZ_{observed} = s(p - pw + qv)^2 / c$ (6)

Heterozygous: $HTZ_{observed} = 2hs(q + pw - qv)(p - pw + qv) / c$

and: $p'_{observed} = \{s(p - pw + qv)^2 + hs(q + pw - qv)(p - pw + qv)\} / c$

So that, under HWE, the expected homozygous frequency would be:

$$HZ_{expected} = (p'_{observed})^2 = [\{s(p - pw + qv)^2 + hs(q + pw - qv)(p - pw + qv)\} / c]^2 \quad (7)$$

Using Definition (5), a simple rearrangement of Equation (7) yields:

$$HZ_{expected} = \{s(p - pw + qv)^2 / c)\} \{1 + (h^2 s - 1)(q + pw - qv)^2 / c\} \quad (8)$$

From Equations (6) and (8), clearly, the value of the quantity $(h^2 s)$ will determine the population status.

Thus, if: $h^2 s < 1$; then both homozygote frequencies will be more than HWE expectations; i.e., Equation (6) > Equation (8)

if: $h^2 s = 1$; then the population is at HWE; i.e., Equation (6) = Equation (8)

and if: $h^2 s > 1$; then both homozygote frequencies will be less than HWE expectations; i.e., Equation (6) < Equation (8)

Several features of this system are evident from a consideration of Equations (6 & 8). First, because, by definition, $(s \leq 1)$, then it must be the case that (if: $h < 1$; then: $h^2 s < 1$). Second, if (p) remains stable at ~10%, then as (v) increases for any given value of $h^2 s$ (except when: $h^2 s = 1$), the magnitude of the deviation from HWE becomes larger. Third, if "*frequent*" CEHs are dominant and only genotypes with homozygous "*rare*" CEHs undergo selection (i.e., when: $h = 1/s$), then $(h^2 s = 1/s \geq 1)$ and, thus, the homozygous frequencies will be equal to or less than their HWE expectations. Fourth, if heterozygotes are less disadvantaged than homozygotes (i.e., if: $h > 1$), then, as (h) increases for any given value of (s), the difference between the observed homozygous frequencies and HWE expectations become smaller until the point at which $(h^2 s = 1)$. After this point, as (h) increases still further, the difference between observation and HWE expectations becomes more negative.

Fifth, because haplotype conversion, by itself, doesn't affect whether or not the population is at HWE (see above), any discrepancy between observation and HWE expectations

will be driven by selection – a circumstance that is reflected by the sole dependence of population status on the quantity $(h^2 s)$ – see Equation (8) above.

We define: $$\Delta(p')^2 = HZ_{observed} - HZ_{expected} = HZ_{observed} - (p'_{observed})^2$$

And we note that, in order for the "*rare*" haplotype frequency *(p)* to remain stable in the population over time requires the condition that: $p'_{observed} = p$

In the WTCCC, the observed homozygous frequencies $(HZ_{observed})$ exceeded the HWE expectations and, specifically for (*p*), it was found that:

$$HZ_{observed} = s(p + qv - pw)^2 / c = 0.014$$

$$(p'_{observed})^2 = (p)^2 = 0.010$$

so that: $$\Delta(p')^2 = 0.004 \qquad (z = 6.7;\ p < 10^{-10})$$

Analyzing Controls and Cases separately yields similar estimates (see below).
Moreover, plausible ranges (i.e., the 95% confidence intervals) can be assigned to these parameters as:

$$0.0126 \leq HZ_{observed} \leq 0.0153$$

$$0.009 \leq (p)^2 \leq 0.011$$

$$0.0021 \leq \Delta(p')^2 \leq 0.0056$$

Using these estimates, together with the estimate of:

$$v \approx 5.4w$$

and by varying the parameter combinations $(\text{when: } h = 1)$ such that:

$$0.1 \leq s \leq 1 \ ; \ \text{ and: } \ 0 \leq v \leq 0.08$$

leads to a solution space for this system consisting of:

$$0.54 \leq s \leq 0.79 \ ; \ \text{ and: } \ 0.0275 \leq v \leq 0.06$$

These limits are unchanged, even when letting: $w \to 0$; (or as: $v / w \to \infty$)

The EPIC data basically confirms these observations. Naturally, because of its much smaller sample size, CEHs with a single representation in EPIC have a predicted frequency more than 30 times greater than CEHs with a single representation in the WTCCC. It is not surprising, therefore, that the group of CEHs, which had only a single representation in EPIC, had a much greater likelihood of occurrence than did the group of single CEHs in the WTCCC. Moreover,

again not surprisingly, many of CEHs with only a single representation in EPIC had multiple representations in the WTCCC. Consequently, in order to identify more accurately the truly unique haplotypes within the EPIC dataset, we analyzed only those CEHs in EPIC, which had a single representation in the combined dataset of the WTCCC and EPIC. Once again, there was a significant over-representation of homozygous "*rare*" genotypes $(z = 2.6)$. In addition, the solution space derived from EPIC observations overlapped that estimated from the WTCCC (see above), although, naturally, the limits were broader because of the smaller sample size.

Consequently, the WTCCC observations imply a remarkably strong selection pressure $(s \approx 0.54 - 0.79)$ against "*rare*" CEHs and, in addition, they also imply a net haplotype conversion rate of approximately 3−6%, in order for "*frequent*" CEHs to remain in the population with a frequency of anything approaching 90%. Because the total genomic distance spanned by these CEHs is approximately 2.7 mb, this implies a net haplotype conversion rate of 1.1−2.2 conversions per mb. Despite the fact that, potentially, there could be as many as 2 haplotype conversions per crossover, this estimated rate still seems somewhat high compared to the reported crossover rate of ~0.4 CM/mb in this genomic region [27, 34, 37, 38]. Nevertheless, the presence of recombination "hot-spots" within the MHC and/or differences in recombination rates between men and women or among individuals and populations may contribute to the occurrence of net conversions [42, 43].

Naturally, if $(h \neq 1)$ the solution space changes. As (h) becomes increasingly greater than one, (s) becomes smaller (i.e., more selection) and (v) becomes greater (i.e., more conversion). By contrast, as (h) becomes increasingly less than one, (s) becomes greater (i.e., less selection) and (v) becomes smaller (i.e., less conversion). On the one hand, this latter configuration might seem more likely because it implies less extreme levels for both selection pressure and haplotype conversion. On the other hand, however, because, in the model, "*rare*" haplotypes are presumed to be, on average, "less fit" immunologically than "*frequent*" haplotypes, it is hard to rationalize how heterozygotes (with only one "less fit" haplotype) could be at a greater selective disadvantage compared to homozygotes (with two "less fit" haplotypes). This circumstance would be the opposite of over-dominance and, consequently, it seems far more likely that: $(h \geq 1)$.

Importantly, there are possible explanations, other than selection, that might also account for an observed deviation from HWE. For example, genetic drift (which typically takes many generations to act) can certainly cause changes in haplotype frequency and would serve to reduce the heterozygosity of the population (i.e., this is a circumstance in which: $h < 1$). Nevertheless,

even for population sizes of 100, the impact of genetic drift in a single generation (which is what we are observing here) is tiny [45] and seems unable to account for the magnitude of the deviation from HWE that we found. Moreover, all but 2 of the regions participating in the WTCCC had sample-sizes of 400 or more (e.g. *Supplemental S2 Table*).

Alternatively, a deviation from HWE could be accounted for if the WTCCC population is composed of two or more sub-populations, each of which is in HWE but with each sub-population having different haplotype frequencies (*rare vs. frequent*). Such a circumstance would violate the HWE assumption of random mating and would lead to homozygotes being in excess of expectations (as we observed). Moreover, this particular mechanism is widely-recognized to cause such HWE deviations and, indeed, there is no question that the WTCCC is composed of a number of ethnically and geographically different populations, many of which have markedly different CEH compositions (e.g., Figure 4; *Supplemental S2 Table*). Nevertheless, there are several reasons to believe that this simple mechanism is also unlikely to explain our observations. First, in this analysis, the haplotype frequencies are defined, not by the biological nature of the haplotypes themselves, but, rather, only by the frequency of their occurrence in the population. This makes the variability of the exact CEH composition between the different populations largely moot and focuses, rather, on the frequency of occurrence of the so-called "*rare*" haplotypes, regardless of their specific biological properties. Second, diverse and long-separated human populations seem to have very similar distributions of "*frequent*" and "*rare*" haplotypes (*Supplemental Figure B in File S3*). Third, and more to the point, we determined (for each region separately) the proportion of "*rare*" haplotypes and calculated the expected impact that this observed diversity in haplotype frequency would have on HWE for the combined WTCCC. Importantly, the observed diversity of "*rare*" haplotype frequency between regions accounted for only:

$$\Delta(p')^2 = 0.001$$

which is considerably less than the deviation from HWE that we actually observed.

And, finally, although the WTCCC cases are very diverse ethnically and geographically (Figure 4; *Supplemental S2 Table*), the controls are, by contrast, quite homogeneous. Despite this relative homogeneity, however, the controls are, if anything, more extreme in their divergence

from HWE than are the cases. Thus, considering only the WTCCC controls, the above parameter estimates become:

$$HZ_{observed} = s(p+qv-pw)^2 / c = 0.016$$

$$(p)^2 = 0.011$$

so that: $$\Delta(p')^2 = 0.005 \qquad (z = 6.3;\ \ p < 10^{-9})$$

And considering only the cases:

$$HZ_{observed} = s(p+qv-pw)^2 / c = 0.010$$

$$(p)^2 = 0.008$$

so that: $$\Delta(p')^2 = 0.002 \qquad (z = 2.4;\ \ p < 0.02)$$

Such findings suggest that the mixing of different populations with varying haplotype frequencies is not sufficient to account for our observations. Rather, the observed deviations from HWE seem more likely to be the combined result of both haplotype conversion and haplotype selection – each a process that takes place in every generation.

Finally, because, the observed deviations from HWE are equal in magnitude for both the homozygous "*rare*" and homozygous "*frequent*" genotypes, it is not possible (from considering exclusively this analysis) to be sure *a priori* that the direction of selection is in favor of the "*frequent*" genotypes as is assumed by Equation (5) above. Nevertheless, when (*p*) and (*q*) are interchanged in this analysis (thereby making selection operate in favor of the "*rare*" haplotypes), the solution space when $(h = 1)$ is essentially confined to the circumstance in which there is no selection and no haplotype conversion. All other solutions in this circumstance require $(h < 1)$, which, as discussed earlier, seems inherently unlikely.

Moreover, as noted previously, in the circumstance of no conversion and no selection, HWE should be attained in, at most, 2 generations [45] and it would have to be viewed as simply coincidental that the cross-sectional WTCCC and EPIC cohorts happened to catch generations in transition. Consequently, as expected, it seems that the effect of selection must be to favor the "*frequent*" haplotypes, as is indicated by Equation (5). In fact, this exact same conclusion is also indicated (even more strongly) simply by the occurrence of a very small number of very common CEHs in the WTCCC, the EPIC, and other populations (Figure 3; *Supplemental Figure C in File S3*, *Supplemental Material #1 in File S1*; *Supplemental S1 Table*).
