# Supplementary material for: Highly conserved extended haplotypes of the major histocompatibility complex and their relationship to multiple sclerosis susceptibility
Source: PLoS One. 2018 Feb 13;13(2):e0190043. doi: 10.1371/journal.pone.0190043 (PMC5810982; doi:10.1371/journal.pone.0190043)
Supplement: S3 File — (PDF) [file pone.0190043.s003.pdf]

# *Supplemental Figures*

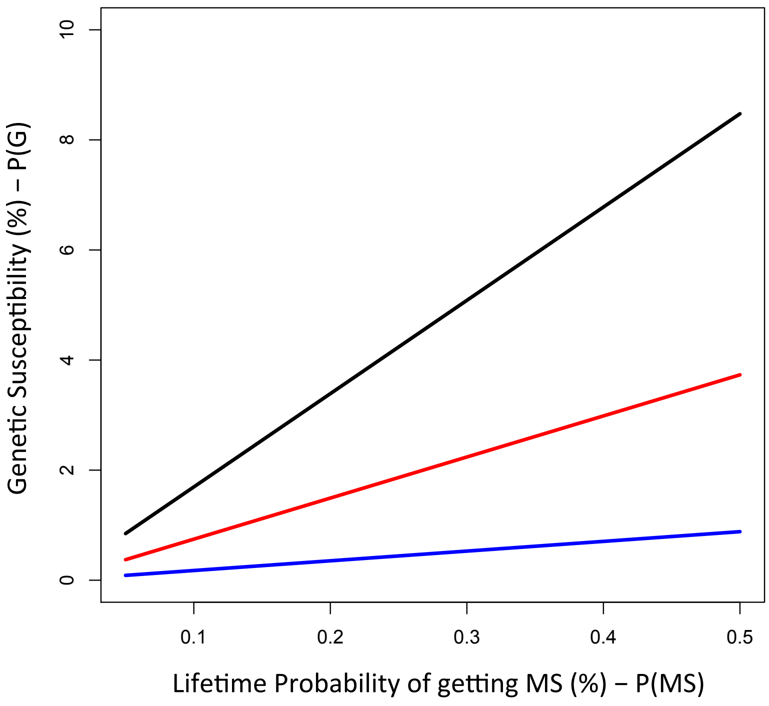


**Supplemental Fig A.** The expected lifetime probability of getting MS for the entire population will be labeled as $P(MS)$, as in Reference [3]. Within this population, we can define the "genetically susceptible" subset ($G$) as all of those ($i$) individuals, each with a unique genotype ($G_i$), for whom the conditional probability $P(MS \mid G_i) \geq P(MS)$. The *x-axis* spans the entire plausible range (0.05–0.5%) for $P(MS)$; a range-estimate, which is based upon numerous published epidemiological reports [3]. The *y-axis* represents the percentage of the population that is in the "genetically susceptible" subset – $P(G)$ – using different assumptions. The equation governing this system [3] is:

$$\{P(G \mid MS)_{min}\} * \{P(MS)_{min} \;/\; P(MS \mid G)_{max}\} \leq P(G) \leq P(MS)_{max} \;/\; P(MS \mid G)_{min}$$

The black line represents the maximum possible percentage for $P(G)$ under those circumstances, in which the conditional probability – $P(MS \mid G)$ – takes on its minimum plausible value (5.9%) [3]. In this case, $P(G)$ reaches its maximum plausible value (8.5%) when $P(MS)$ is at the top of

its plausible range. The blue line represents the minimum possible percentage for $P(G)$ under those circumstances in which the conditional probability $- P(G \mid MS) -$ takes on its minimum plausible value (84%) and the conditional probability $- P(MS \mid G) -$ takes on its maximum plausible value (48%) [3]. The red line represents the expected value of $P(G)$, which is estimated from observed recurrence risks of MS in identical twins (25%), fraternal twins (5.4%) and siblings (2.9%), as reported in several population-based studies from Canada [3]. Consequently, with 24% of the population being *HLA-DRB1*15:01~HLA-DQB1*06:02~a1* carriers, with only half of MS patients carrying this haplotype, and with fewer than 8.5% of the population being "genetically susceptible" to getting MS, it is clear that the large majority of *HLA-DRB1*15:01~HLA-DQB1*06:02~a1* carriers cannot even be within the "genetically susceptible" subset [3].

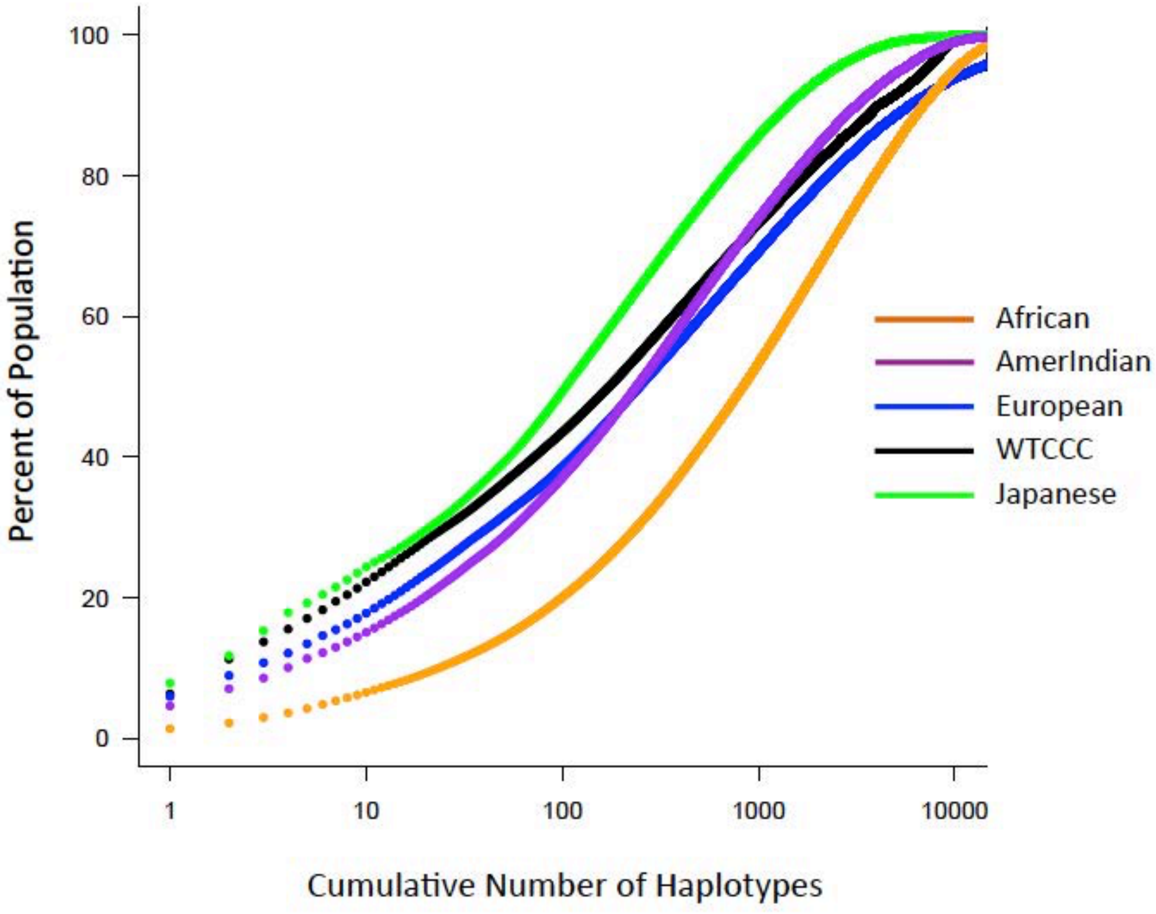


**Supplemental Fig B.** All haplotypes (CEHs) in the WTCCC dataset and the Gragert et al. [35] study datasets for Africans, Europeans, AmerIndians, and Japanese were sorted by descending haplotype frequency within their respective populations. This graph plots the cumulative number of unique haplotypes (beginning with the highest frequency haplotype) in each population against the percentage of the total number of haplotypes for each of these populations. The large majority of the unique haplotypes in all the different populations have only a very small number of representations (low-frequency), whereas the majority of the total haplotypes are accounted for by only a very small number of very high-frequency unique haplotypes (see also: Figure 3, *Main Text*).

**Haplitypes containing HLA DRB1-1501**

**Haplitypes containing HLA DRB1-1501**

| Name | c2 | c3 | c6 | c11 | c13 | c16 | c19 | c22 | c28 | c44 | c50 | c58 | c78 | c87 | c91 | c108 | c116 | c120 | c125 | c128 | c132 | c139 | c140 | c143 | c173 |
|---|---|---|---|---|---|---|---|---|---|---|---|---|---|---|---|---|---|---|---|---|---|---|---|---|---|
| c3 | 5.1 | | | | | | | | | | | | | | | | | | | | | | | | |
| c6 | * | -2.4 | | | | | | | | | | | | | | | | | | | | | | | |
| c11 | * | -4.5 | -2.3 | | | | | | | | | | | | | | | | | | | | | | |
| c13 | * | -3.3 | * | * | | | | | | | | | | | | | | | | | | | | | |
| c16 | * | -3.7 | * | * | * | | | | | | | | | | | | | | | | | | | | |
| c19 | 3.3 | * | * | 3.7 | 2.8 | 3.2 | | | | | | | | | | | | | | | | | | | |
| c22 | * | * | * | 2.3 | * | * | * | | | | | | | | | | | | | | | | | | |
| c28 | * | -3.8 | * | * | * | * | -3.5 | -2.5 | | | | | | | | | | | | | | | | | |
| c44 | * | * | * | * | * | * | * | * | * | | | | | | | | | | | | | | | | |
| c50 | * | * | * | * | * | * | * | * | * | * | | | | | | | | | | | | | | | |
| c58 | * | * | * | * | * | * | * | * | * | * | * | | | | | | | | | | | | | | |
| c78 | * | * | * | * | * | * | -2.0 | * | * | * | * | * | | | | | | | | | | | | | |
| c87 | * | * | * | * | * | * | * | * | * | * | * | * | * | | | | | | | | | | | | |
| c91 | * | * | * | * | * | * | * | * | * | * | * | * | * | * | | | | | | | | | | | |
| c108 | * | * | * | * | * | * | * | * | * | * | * | * | * | * | * | | | | | | | | | | |
| c116 | * | -2.2 | * | * | * | * | -2.2 | * | * | * | * | * | * | * | * | * | | | | | | | | | |
| c120 | * | -2.3 | * | * | * | * | -2.3 | * | * | * | * | * | * | * | * | * | * | | | | | | | | |
| c125 | * | * | * | 2.3 | * | 2.1 | * | * | 2.5 | * | * | * | * | * | * | * | * | 2.0 | | | | | | | |
| c128 | * | * | * | * | * | * | * | * | * | * | * | * | * | * | * | * | * | * | * | | | | | | |
| c132 | * | * | * | 2.3 | * | 2.1 | * | * | * | * | * | * | * | * | * | * | * | 2.1 | * | * | | | | | |
| c139 | * | * | * | * | * | * | * | * | * | * | * | * | * | * | * | * | * | * | * | * | * | | | | |
| c140 | * | * | * | * | * | * | * | * | * | * | * | * | * | * | * | * | * | * | * | * | * | * | | | |
| c143 | * | * | * | * | * | * | * | * | * | * | * | * | * | * | * | * | * | * | * | * | * | * | * | | |
| c173 | * | -2.6 | * | * | * | * | -2.6 | * | * | * | * | * | * | * | * | * | * | * | -2.3 | * | -2.4 | * | * | * | |
| c282 | -3.0 | -3.6 | -3.2 | -2.7 | -2.9 | -2.7 | -3.6 | -3.3 | -2.4 | -3.0 | -2.9 | -2.8 | -2.6 | -2.7 | -3.1 | -2.8 | -2.3 | -2.2 | -3.5 | -2.9 | -3.5 | -2.6 | -2.6 | -2.8 | * |

**Supplemental Fig C.** Z-scores for differences in disease-association odds ratios (ORs) between the different HLA-DRB1*15:01-containing haplotypes presented in Table 2 (*Main Text*). These haplotypes (Names) are listed on both the x-axis (as columns) and y-axis (as rows) and the z-scores for each comparison are represented as numbers at the points of intersection of the column and row for any two haplotypes. Comparisons with a z-score<2.0, are represented by asterisks (*). Positive numbers indicate that the haplotype in the column has a greater OR than the haplotype in the row. Conversely, negative numbers indicate that the haplotype in the column has a smaller OR than the haplotype in the row. For example, haplotype (*c282*) has a significantly larger OR than every other haplotype except for haplotype (*c173*). Similarly haplotype (*c3*) is significantly smaller than a number of other haplotypes.
