# Supplementary material for: Highly conserved extended haplotypes of the major histocompatibility complex and their relationship to multiple sclerosis susceptibility
Source: PLoS One. 2018 Feb 13;13(2):e0190043. doi: 10.1371/journal.pone.0190043 (PMC5810982; doi:10.1371/journal.pone.0190043)
Supplement: S3 Table — (DOCX) [file pone.0190043.s006.docx]

**S3 Table.** Common Extended haplotypes in the EPIC^†^**^†^**

|  |  | | |  | |  | |  | |  |  |
| --- | --- | --- | --- | --- | --- | --- | --- | --- | --- | --- | --- |
| **Name^†^** | **HLA Haplotype**  ***A~C~B~DRB1~DQB1~SNP*** | | | **Frequency** | | **OR^*^** | | | **p-value^**^** | |  |
|  |  | | |  | |  | | |  | |  |
| *c1* | *01:01~07:01~08:01~03:01~02:01~a6* | | 101 | | | 1.3 (0.8 – 2.1) | | | ns | |  |
| *c2* | *03:01~07:02~07:02~15:01~06:02~a1* | | 89 | | | 2.6 (1.5 – 4.7) | | | 0.0002 | |  |
| *c3* | *02:01~07:02~07:02~15:01~06:02~a1* | | 38 | | | 3.2 (1.4 – 7.9) | | | 0.003 | |  |
| *c5* | *02:01~05:01~44:02~04:01~03:01~a3* | | 26 | | | 0.2 (0.0 – 0.6) | | | 0.001 | |  |
| *c11* | *25:01~12:03~18:01~15:01~06:02~a1* | | 21 | | | 3.6 (1.0 – 15.5) | | | 0.03 | |  |
| *c6* | *24:02~07:02~07:02~15:01~06:02~a1* | | 20 | | | 3.4 (0.8 – 20.4) | | | 0.06 | |  |
|  | |  | | |  | |  | | | |  |
|  | |  | | |  | |  | | | |  |

†† *a1* containing haplotypes with ≥ 20 representations in the EPIC.

† Arbitrary name for haplotype (sorted in descending order of frequency) for the EPIC population (see Tables 2 and 3 ; Main Text)

* Odds ratio (OR) of disease for individuals having 1 copy of the listed haplotype compared to having either no, or no other, copies of the *HLA*-*DRB1*15:01~HLA*-*DQB1*06:02~a1* Class II haplotype (95% CI range in parenthesis)

****** Significance of the association between having 1 copy of the specific allele and the disease (MS) compared to having no copies.
